# Phase and structure engineering of copper tin heterostructures for efficient electrochemical carbon dioxide reduction

**DOI:** 10.1038/s41467-018-07419-z

**Published:** 2018-11-22

**Authors:** Pengtang Wang, Man Qiao, Qi Shao, Yecan Pi, Xing Zhu, Yafei Li, Xiaoqing Huang

**Affiliations:** 1 College of Chemistry, Chemical Engineering and Materials Science, Soochow University, 215123 Jiangsu, China; 2 Jiangsu Collaborative Innovation Centre of Biomedical Functional Materials, School of Chemistry and Materials Science, Nanjing Normal University, 210023 Nanjing, China; 3 Testing & Analysis Center, Soochow University, 215123 Jiangsu, China

## Abstract

While engineering the phase and structure of electrocatalysts could regulate the performance of many typical electrochemical processes, its importance to the carbon dioxide electroreduction has been largely unexplored. Herein, a series of phase and structure engineered copper-tin dioxide catalysts have been created and thoroughly exploited for the carbon dioxide electroreduction to correlate performance with their unique structures and phases. The copper oxide/hollow tin dioxide heterostructure catalyst exhibits promising performance, which can tune the products from carbon monoxide to formic acid at high faradaic efficiency by simply changing the electrolysis potentials from −0.7 V_RHE_ to −1.0 V_RHE_. The excellent performance is attributed to the abundant copper/tin dioxide interfaces involved in the copper oxide/hollow tin dioxide heterostructure during the electrochemical process, decreasing the reaction free-energies for the formation of COOH* species. Our work reported herein emphasizes the importance of phase and structure modulating of catalysts for enhancing electrochemical CO_2_ reduction and beyond.

## Abstract (web summary)

While CO_2_ removal will play a crucial role in limiting climate change, it is challenging to understand the factors that control materials’ selectivity to convert CO_2_ to valuable products. Here, authors show copper and tin oxide interfaces to impact activities for CO_2_ reduction products.

## Introduction

The increased CO_2_ level in the atmosphere has caused many climatic concerns, such as global warming, melting glaciers, and more disastrous weather^1^. The conversion of CO_2_ into renewable energy sources has attracted extensive attention; this conversion not only decreases the CO_2_ concentration in the atmosphere but also stores renewable energy^2–5^. Recently, the electroreduction of CO_2_ has attracted particular attention^5,6^, mainly due to its feasible operating conditions and the potential of sustainable direct integration with renewable resources. Many studies have reported the electrochemical CO_2_ reduction reaction (CO_2_RR) by using different catalysts^6,7^. For example, noble catalysts, such as Au^8,9^, Ag^10,11^, and Pd^12^, are generally reported to reduce CO_2_ into CO, whereas main group metals In^13^, Sn^14^, Pb^15^, and Bi^16^ favor the generation of formic acid (HCOOH) during the CO_2_RR. Unlike noble metal and main group metal catalysts, Cu is active to electroreduce CO_2_ into hydrocarbons or oxygenates^17–19^, but it also generates common products, such as CO, HCOO^−^ and H_2_^20,21^. Owing to the unique behavior of Cu in the CO_2_RR, substantial research efforts have been devoted to improve the activity and selectivity of Cu catalysts for the CO_2_RR^22,23^. Nevertheless, the achieved faradaic efficiency (FE) for the useful products is unsatisfactory and usually <40%. To this end, increasingly more researchers focus on combining other metals with Cu to achieve bimetallic Cu-based catalysts^13,24^, which could bring extra synergistic effects or stabilize the reaction intermediates to improve the activity and selectivity of the CO_2_RR^25–28^. Recently, relevant works, such as the synthesis of CuAu^25^, CuPd^26^, and CuSn^27,28^ bimetallic catalysts, etc. have been intensively reported, showing the enhanced performance for the CO_2_RR^25–28^. However, it should be pointed out that, while the majority of these previous studies focus on improving the CO_2_RR selectivity for only one product, the selectivity for different useful products at high levels cannot be tuned. In addition, most of these reports are limited to studying the composition (ratio of two metals) effect for the CO_2_RR, while the structure and phase effects of bimetallic catalysts on the CO_2_RR are largely unexplored.

It is well known that the bimetallic-based catalyst structures (e.g., core–shell structures, heterostructures, alloying structures, etc.) or phases (e.g., metal/metal phases, metal/oxide phases, metal/sulfide phases, etc.) usually have significant influences on their electrochemical performance^29^. Such observations have been demonstrated in several electrochemical processes, such as the hydrogen evolution reaction (HER) and the oxygen reduction reaction^30–32^, while limited studies have revealed the structure and phase effects on the CO_2_RR. Herein, by taking advantage of the intrinsic chemical reactivity differences of the bimetallic components, we successfully develop three distinct Cu–SnO_2_ bimetallic catalysts with different structures and phases: the CuO/hollow SnO_2_ heterostructure of CuSn nanoparticles (NPs)/C-A, the Cu_41_Sn_11_@SnO_2_ core–shell structure of CuSn NPs/C-H, and the Cu NPs/hollow SnO_2_ Janus structure of CuSn NPs/C-AH through a simple yet efficient post-annealing treatment of CuSn core–shell NPs at controlled conditions. These Cu–SnO_2_ bimetallic catalysts are thoroughly exploited for the electrochemical CO_2_RR, and the CO_2_RR performance is correlated with their unique structures and phases. This reveals that the optimized CuSn NPs/C-A exhibits promising activity and selectivity for the electrochemical CO_2_RR, which can tune the product from CO to HCOOH at high FE values by simply changing the electrolysis potential from −0.7 to −1.0 V_RHE_. Moreover, it also exhibits the highest CO_2_RR activity in all the Cu–SnO_2_ bimetallic catalysts, where the partial current densities of CO at −0.7 V_RHE_ are 5.9 and 5.3 times higher and of HCOOH at −1.0 V_RHE_ are 33.2 and 3.4 times higher than those of the individual Cu NPs/C and SnO_2_ nanoshell/C (SnO_2_ NSL/C), respectively. We attribute the excellent CO_2_RR performance to its abundant Cu/SnO_2_ interfaces involved in the CuO/hollow SnO_2_ heterostructure during the electrochemical process, which decrease the reaction free-energies (Δ*G*) for the formation of COOH* species. Furthermore, under a long-term durability test, these Cu–SnO_2_ bimetallic catalysts can endure at least 10 h with small activity and selectivity decays.

## Results

### Preparation and characterization of CuSn NPs

**Fig. 1:**
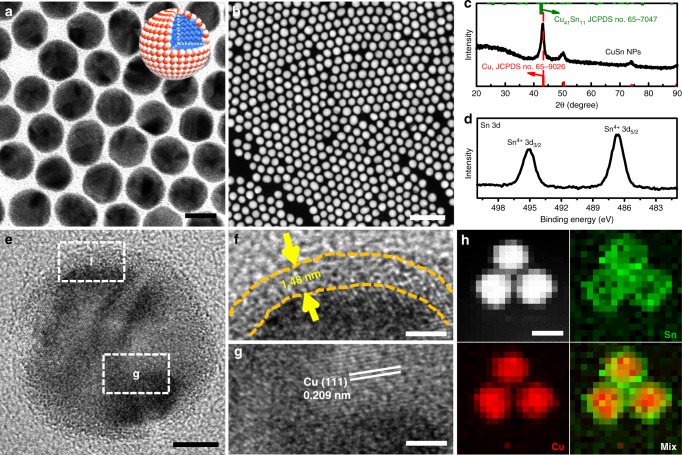
Structure and phase characterization of CuSn NPs. **a** Transmission electron microscopic (TEM) image and atom model, **b** high-angle annular dark-field scanning TEM (HAADF-STEM) image, **c** X-ray diffraction (XRD) pattern, and **d** Sn 3d X-ray photoelectron spectroscopy (XPS) pattern of CuSn NPs. **e** High-resolution TEM (HRTEM) image and **f**, **g** the magnified regions in **e** of CuSn NPs. **h** HAADF-STEM and energy-dispersive X-ray spectroscopy (EDS) elemental mapping images of CuSn NPs. Scale bars, 2 nm in **f**, **g**, 5 nm in **e**, 20 nm in **a**, **h**, 100 nm in **b**. Red, blue, and white spheres in the model represent O, Cu, and Sn atoms, respectively

The CuSn NPs were obtained via the co-reduction of copper (II) acetylacetonate (Cu(acac)_2_), and dibutyltin bis(2,4-pentanedionate) (C_18_H_32_O_4_Sn) at 180 °C for 3 h with ascorbic acid (AA) as the reducing agent and oleylamine (OAm) as the solvent and surfactant. Transmission electron microscopy (TEM) (Fig. 1a) and high-angle annular dark-field scanning TEM (HAADF-STEM) images (Fig. 1b) clearly show that the CuSn NPs are highly dispersive. The diameter of the CuSn NPs was measured to be 23.0 ± 3.5 nm (Supplementary Fig. 1). The Cu/Sn ratio of the CuSn NPs was determined to be 80.7/19.3 by scanning electron microscopy energy-dispersive X-ray spectroscopy (SEM-EDS) (Supplementary Fig. 2). Powder X-ray diffraction (XRD) (Fig. 1c) was carried out to study the phase of the CuSn NPs; the diffraction peaks are perfectly indexed to face-centered cubic Cu, suggesting that the component of Sn are likely amorphous in CuSn NPs. The X-ray photoelectron spectroscopy (XPS) pattern was further carried out to confirm the surface environment of the CuSn NPs, where the Sn 3d_5/2_ and 3d_3/2_ peaks of CuSn NPs are located at 486.6 and 495.0 eV, respectively, which can be assigned to the 3d_5/2_ and 3d_3/2_ peaks of Sn^4+^ (Fig. 1d)^28^, confirming that the Sn species in CuSn NPs is present in the form of amorphous SnO_2_. To further investigate the distribution of amorphous SnO_2_ in CuSn NPs, high-resolution TEM (HRTEM) for a single CuSn NP was carried out. As shown in Fig. 1e, it is obvious that there is an amorphous SnO_2_ shell surrounding the crystalline Cu core. Figure 1f, g display the magnified images recorded from regions f and g marked in Fig. 1e, where the thickness of amorphous SnO_2_ shell is approximately 1.48 nm, consistent with the HAADF-STEM line scan result of 1.43 nm (Supplementary Fig. 3). The interplanar spacing of the lattice fringe was measured to be 0.209 nm, consistent with the (111) plane of metallic Cu (Fig. 1g). The distributions of Sn and Cu in CuSn NPs were further confirmed by HAADF-STEM EDS elemental mappings (Fig. 1h), where Cu is limited to the core of the CuSn NPs, while Sn surrounds the Cu core. Hence, we can conclude that the prepared CuSn NPs have a core–shell structure with metallic Cu cores and amorphous SnO_2_ shells.

### Preparation and characterization of CuSn NPs/C-A, CuSn NPs/C-H, and CuSn NPs/C-AH

**Fig. 2:**
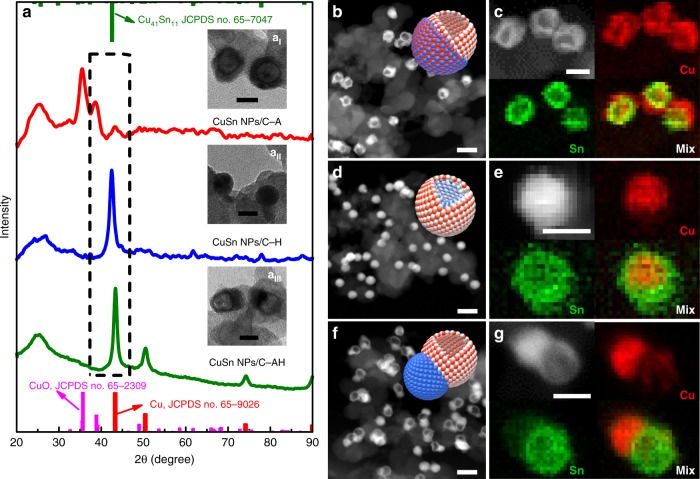
Structure and phase characterizations of CuSn NPs/C with controlled annealing treatment. **a** X-ray diffraction (XRD) patterns and inset transmission electron microscopic (TEM) images of **a**_**I**_ CuSn NPs/C-A, **a**_**II**_ CuSn NPs/C-H, and **a**_**III**_ CuSn NPs/C-AH. **b**, **d**, **f** High-angle annular dark-field scanning TEM (HAADF-STEM) images and atom models and **c**, **e**, **g** HAADF-STEM images and energy-dispersive X-ray spectroscopy (EDS) elemental mappings of **b**, **c** CuSn NPs/C-A, **d**, **e** CuSn NPs/C-H, and **f**, **g** CuSn NPs/C-AH. Scale bars, 50 nm in **b**, **d**, **f**, 20 nm in **a**_**I**_–**a**_**III**_, **c**, **e**, **g**. Red, blue, and white spheres in the models represent O, Cu, and Sn atoms, respectively

Considering that the chemical reactivities of SnO_2_ and Cu are quite different, the thermal annealing treatment is expected to be effective to induce phase and structure changes of the pristine CuSn NPs. We thus focus on the phase and structure engineering of CuSn NPs through annealing. To this end, the pristine core–shell CuSn NPs were loaded on a carbon (C, VC-X72) by sonication, denoted as CuSn NPs/C. The core–shell CuSn NPs are highly dispersive on C, without shape, size, phase, and composition changes after the sonication process (Supplementary Fig. 4). The CuSn NPs/C was then annealed at three controlled conditions. The CuSn NPs/C samples treated at 250 °C for 1 h in air and H_2_/N_2_ (5/95) were denoted as CuSn NPs/C-A and CuSn NPs/C-H, respectively. The CuSn NPs/C-A sample was further annealed in H_2_/N_2_ (5/95) at 250 °C for another 1 h, which was denoted as CuSn NPs/C-AH. As shown in Supplementary Fig. 5, the EDS results reveal that the Cu/Sn ratios in CuSn NPs/C-A, CuSn NPs/C-H, and CuSn NPs/C-AH are 80.3/19.7, 79.3/20.7, and 79.2/20.8, respectively, suggesting that the thermal annealing treatment hardly induced composition changes. XRD was further carried out to analyze the structures after different annealing treatments. It is confirmed that the primary diffraction peaks of Cu largely decreased in the CuSn NPs/C-A with the appearance of distinct diffraction peaks of CuO (Fig. 2a), suggesting that the phase of CuSn NPs/C-A was mainly transformed into CuO after annealing in air. As presented in Supplementary Fig. 6, the CuSn NPs/C-H was transformed into the Cu_41_Sn_11_ alloy, as observed in the enlarged XRD pattern between 37.0° and 47.0°. By further reannealing the CuSn NPs/C-A at 250 °C for 1 h in H_2_/N_2_ (5/95) (CuSn NPs/C-AH), the CuO phase was transformed into metallic Cu. It is worth mentioning that the diffraction peaks of SnO_2_ do not appear throughout the different annealing treatments, suggesting that such annealing treatment cannot crystallize the amorphous SnO_2_.

Besides the phase structure, the chemical states of CuSn NPs/C, CuSn NPs/C-A, CuSn NPs/C-H, and CuSn NPs/C-AH were further investigated by XPS and Auger spectra. Supplementary Fig. 7a shows the XPS pattern of Cu 2p, in which the peaks located at 952.2 and 932.3 eV belong to Cu^0,1+^ species, whereas the peaks at 953.8 and 933.8 eV belong to Cu^2+^ species^28^. The Cu in the initial CuSn NPs/C is in both Cu^0,1+^ and Cu^2+^ states. By being heated in air or H_2_/N_2_ (5/95), Cu^0,1+^ species in CuSn NPs/C-A disappeared, while the proportion of the Cu^0,1+^ species increased in CuSn NPs/C-H. After reannealing the CuSn NPs/C-A at 250 °C in H_2_/N_2_ (5/95) for another 1 h, Cu^0,1+^ species reappeared in the CuSn NPs/C-AH. The Auger spectra of Cu L_3_M_45_M_45_ was further carried out to distinguish the Cu^0^ and Cu^1+^ in these catalysts (Supplementary Fig. 8). In general, the characteristic peak of Cu^0^ for Cu L_3_M_45_M_45_ is located from 918.2 to 918.6 eV, whereas for Cu^1+^ is between 916.0 and 916.4 eV and the peak for the standard Cu^2+^ is from 917.6 to 917.8 eV^33^. The CuSn NPs/C-A shows a peak at 917.6 eV, confirming that there is Cu^2+^ in the CuSn NPs/C-A. The CuSn NPs/C-H and CuSn NPs/C-AH all have peaks at 916.8 eV and 918.4 eV, suggesting that both Cu^0^ and Cu^1+^ are present in the CuSn NPs/C-H and CuSn NPs/C-AH. The binding energy of Sn 3d for these catalysts was also explored (Supplementary Fig. 7b); the chemical state of Sn for all catalysts was mainly SnO_2_, while the binding energy of Sn in CuSn NPs/C-H and CuSn NPs/C-AH shifted to the high level of 487.0 and 486.9 eV compared to the initial CuSn NPs/C and CuSn NPs/C-A (486.6 eV), likely due to the charge transfer of SnO_2_ by the reduction of H_2_. Therefore, we can conclude that the phase of CuSn NPs/C has been readily tuned by thermal annealing in controlled conditions.

The electron microscopy characterizations were further utilized for investigating the structure change of CuSn NPs/C after thermal annealing at different conditions. Fig. 2a_I_, b show the TEM and STEM images of CuSn NPs/C-A. Interestingly, the initial solid CuSn NPs are transformed into hollow structures, with the size increasing to 26.5 ± 3.5 nm (Supplementary Fig. 9a-b). The HAADF elemental mappings further reveal that the Sn matches well with the hollow shell accompanied by Cu on its surface (Fig. 2c), indicating that the interior Cu diffuses outside to adhere to the hollow SnO_2_ shell due to the different diffusion rates of Cu and Sn at the core/shell boundary under the annealing treatment in air^34^. The HRTEM image of CuSn NPs/C-A further shows that the lattice fringe of the section adhering on the SnO_2_ shell is 0.251 nm, which is the (−111) plane of CuO (Supplementary Fig. 10a, b). Hence, the CuSn NPs/C-A has evolved into a CuO/hollow SnO_2_ heterostructure. In contrast, the structure of CuSn NPs/C-H is largely maintained compared to the initial CuSn NPs/C (Fig. 2a_II_, d, e and Supplementary Fig. 9c, d), while the interplanar spacing of the core of CuSn NPs/C-H is 0.212 nm, which is the (660) plane of intermetallic Cu_41_Sn_11_ (Supplementary Fig. 10c, d), revealing that the CuSn NPs/C-H has a core–shell structure. Impressively, after reannealing the CuSn NPs/C-A at 250 °C in H_2_/N_2_ (5/95) for another 1 h, H_2_ transformed CuO into metallic Cu conjoined with the hollow SnO_2_ shell to form a Janus structure with the increased size of 32.5 ± 4.5 nm (Fig. 2a_III_, f and Supplementary Fig. 9e, f). The HRTEM and HAADF elemental mappings further confirm the Janus structure of CuSn NPs/C-AH, with the Cu NP as one side and the hollow SnO_2_ as the other side (Fig. 2g and Supplementary Fig. 10e, f). Therefore, it can be concluded that, after the phase and structure engineering by the simple thermal annealing treatment in controlled conditions, we have successfully transformed the initial core–shell CuSn NPs into three distinct catalysts with different phases and structures: the CuO/hollow SnO_2_ heterostructure of CuSn NPs/C-A, the Cu_41_Sn_11_@SnO_2_ core–shell structure of CuSn NPs/C-H, and the Cu NPs/hollow SnO_2_ Janus structure of CuSn NPs/C-AH. All the structures are presented by their corresponding models in the inset in Fig. 2b, d, f.

### CO_2_RR performances of CuSn NPs/C-A, CuSn NPs/C-H, and CuSn NPs/C-AH

**Fig. 3:**
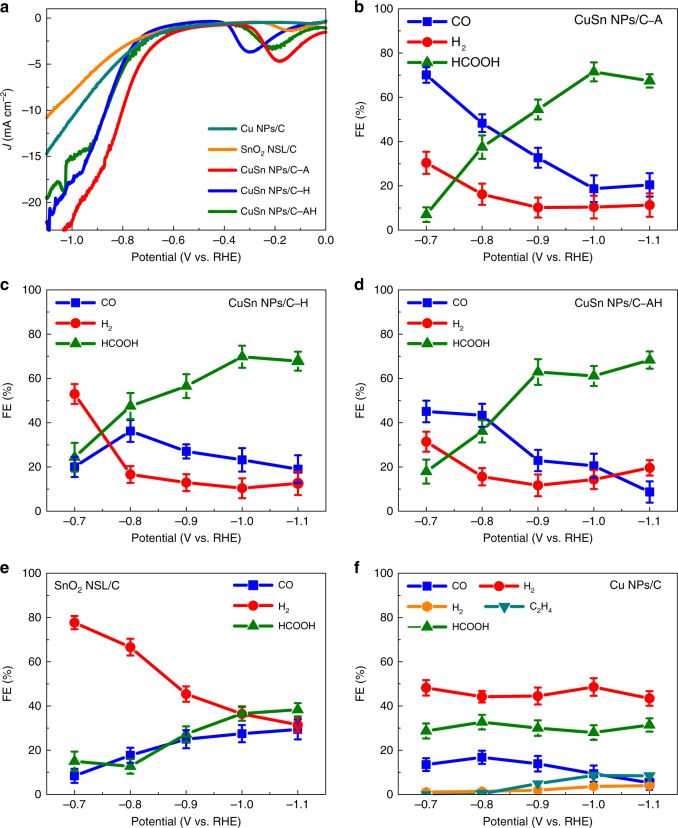
Electrochemical CO_2_ reduction performance of various Cu–SnO_2_ catalysts, Cu NPs/C and SnO_2_ NSL/C. **a** Linear sweeping voltammetric curves of electrochemical CO_2_ reduction measured in 0.1 M KHCO_3_ for different catalysts. Scan rate: 5 mV s^−1^. Reduction potential-dependent faradaic efficiencies (FEs) of H_2_, CO, and HCOOH for **b** CuSn NPs/C-A, **c** CuSn NPs/C-H, **d** CuSn NPs/C-AH, **e** SnO_2_ NSL/C, and **f** Cu NPs/C

Considering that Cu and Sn are believed to be the active components in the CO_2_RR^14,17–21^, the structure and phase effect of the CuSn NPs/C on the CO_2_RR deserves further investigation. To this end, the CO_2_RR performances of the CuSn NPs/C-A, CuSn NPs/C-H, and CuSn NPs/C-AH were fully evaluated in 0.1 M KHCO_3_ (Fig. 3). To reveal the bimetallic effect, the Cu NPs/C and SnO_2_ NSL/C were prepared as references via a solvothermal method and chemical etching of CuSn NPs/C-A, respectively (Supplementary Fig. 11-12). The CO_2_RR performance was measured by the chronoamperometry method under different potentials (Supplementary Fig. 13). The products in gas and liquid phase were analyzed via gas chromatography (GC) and nuclear magnetic resonance (NMR), respectively (Supplementary Fig. 14-15). Figure 3a shows the polarization curves of CO_2_RR using linear sweeping voltammetric methods at 5 mV s^−1^. Compared to the SnO_2_ NSL/C and Cu NPs/C, all the Cu–SnO_2_ bimetallic catalysts with different phases and structures exhibit relative high activity from −0.7 to −1.1 V_RHE_, suggesting that the combination of Cu and Sn enhances the electrocatalytic response. Figure 3b, f show potential-dependent FEs of the major products for CO_2_RR by using different catalysts. The electrocatalytic process mainly produces H_2_, CO, and HCOOH from −0.7 to −1.1 V_RHE_ as well as CH_4_ and C_2_H_4_ at −1.0 and −1.1 V_RHE_ for all catalysts except SnO_2_ NSL/C (Supplementary Fig. 16). The Cu–SnO_2_ bimetallic catalysts exhibit lower FE_H2_ below −0.7 V_RHE_ compared with Cu NPs/C and SnO_2_ NSL/C, revealing that the Cu–SnO_2_ bimetallic catalysts are propitious to depress the HER during the CO_2_RR. Moreover, the product FEs of Cu–SnO_2_ bimetallic catalysts present a trend as the potentials change from −0.7 to −1.0 V_RHE_; FE_HCOOH_ gradually increases along with FE_CO_ and FE_H2_ decrease, which is different from that of Cu NPs/C or SnO_2_ NSL/C, revealing that the combination of Cu and SnO_2_ affects the product selectivity of the CO_2_RR.

**Fig. 4:**
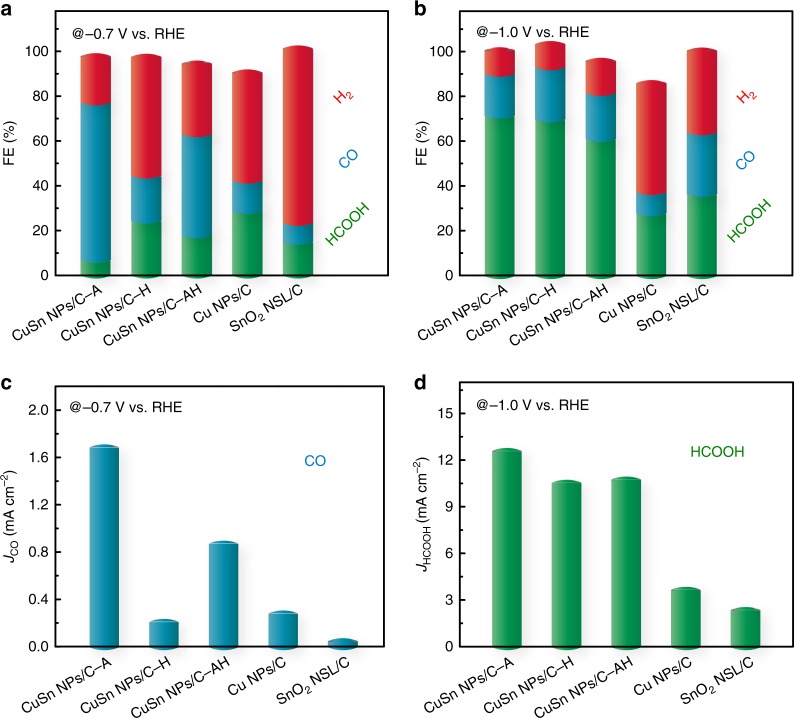
Comparison of the faradaic efficiencies (FEs) and partial current densities of the electrochemical CO_2_ reduction products for various catalysts under specific potential. Histograms of CO, H_2_ and HCOOH FEs at **a** −0.7 V_RHE_ and **b** −1.0 V_RHE_ for different catalysts. Histograms of current densities of **c** CO at −0.7 V_RHE_ and **d** HCOOH at −1.0 V_RHE_ for different catalysts

To further determine the correlation between CO_2_RR performance and their structures and phases, we summarized the FEs of H_2_, CO, and HCOOH for these five catalysts at −0.7 and −1.0 V_RHE_ in Fig. 4a, b, respectively. It clear shows that the main product of the CO_2_RR was CO under the potential of −0.7 V_RHE_ for CuSn NPs/C-A (70.1% FE_CO_) and CuSn NPs/C-AH (45.1% FE_CO_), which largely surpassed the CuSn NPs/C-H (20.1% FE_CO_), Cu NPs/C (13.5% FE_CO_), and SnO_2_ NSL/C (8.4% FE_CO_). Moreover, at −0.7 V_RHE_ the CuSn NPs/C-A reached the maximum partial current density of CO (1.66 mA cm^−2^, Supplementary Fig. 17), which was 1.9, 7.9, 5.9, and 33.2 times higher than the values of the CuSn NPs/C-AH (0.86 mA cm^−2^), CuSn NPs/C-H (0.21 mA cm^−2^), Cu NPs/C (0.28 mA cm^−2^), and SnO_2_ NSL/C (0.05 mA cm^−2^), respectively (Fig. 4c). The FE and partial current density for the CO under −0.7 V_RHE_ of the CuSn NPs/C-A surpass the reported oxidized-derived copper catalysts^6,33,35^, the home-made oxidized-derived copper (CuO NPs/C and Cu_2_O NPs/C catalysts) (Supplementary Fig. 18 and Supplementary Fig. 19), and even comparable to those reported Au^8^, Ag^11^, and Pd^12^ catalysts. When the potential applied was −1.0 V_RHE_, the FEs of CO and H_2_ sharply decreased, with HCOOH as the main product for all catalysts except for Cu NPs/C. Particularly, the CuSn NPs/C-A still achieved the highest FE_HCOOH_ of 71.5%, successfully realizing the tuning of the product FE from CO to HCOOH at ~70% by simply changing the electrolysis potential. Such tunable selectivity of CO and HCOOH at high levels surpassed most reported Cu-based bimetallic CO_2_RR catalysts (Supplementary Table 1). Meanwhile, at −1.0 V_RHE_ the CuSn NPs/C-A also exhibited the highest partial current density of HCOOH (12.6 mA cm^−2^, Fig. 4d), which exceeded the CuSn NPs/C-AH (10.8 mA cm^−2^) and CuSn NPs/C-H (10.5 mA cm^−2^) and was far better than the Cu NPs/C and SnO_2_ NSL/C. Finally, the long-term stabilities of CuSn NPs/C-A, CuSn NPs/C-H, and CuSn NPs/C-AH were also investigated using chronoamperometry at −0.7 V_RHE_. As shown in Supplementary Fig. 20, all the above catalysts achieved stable current densities in the span of 10 h. Meanwhile, the product FE values of these catalysts could also be maintained at the initial value, suggesting that the CO_2_RR performances of these Cu–SnO_2_ bimetallic catalysts is stable. Furthermore, the hollow SnO_2_ shell and heterostructure of CuSn NPs/C-A were largely maintained after the stability test, revealing the stable structure of CuSn NPs/C-A (Supplementary Fig. 21).

## Discussion

**Fig. 5:**
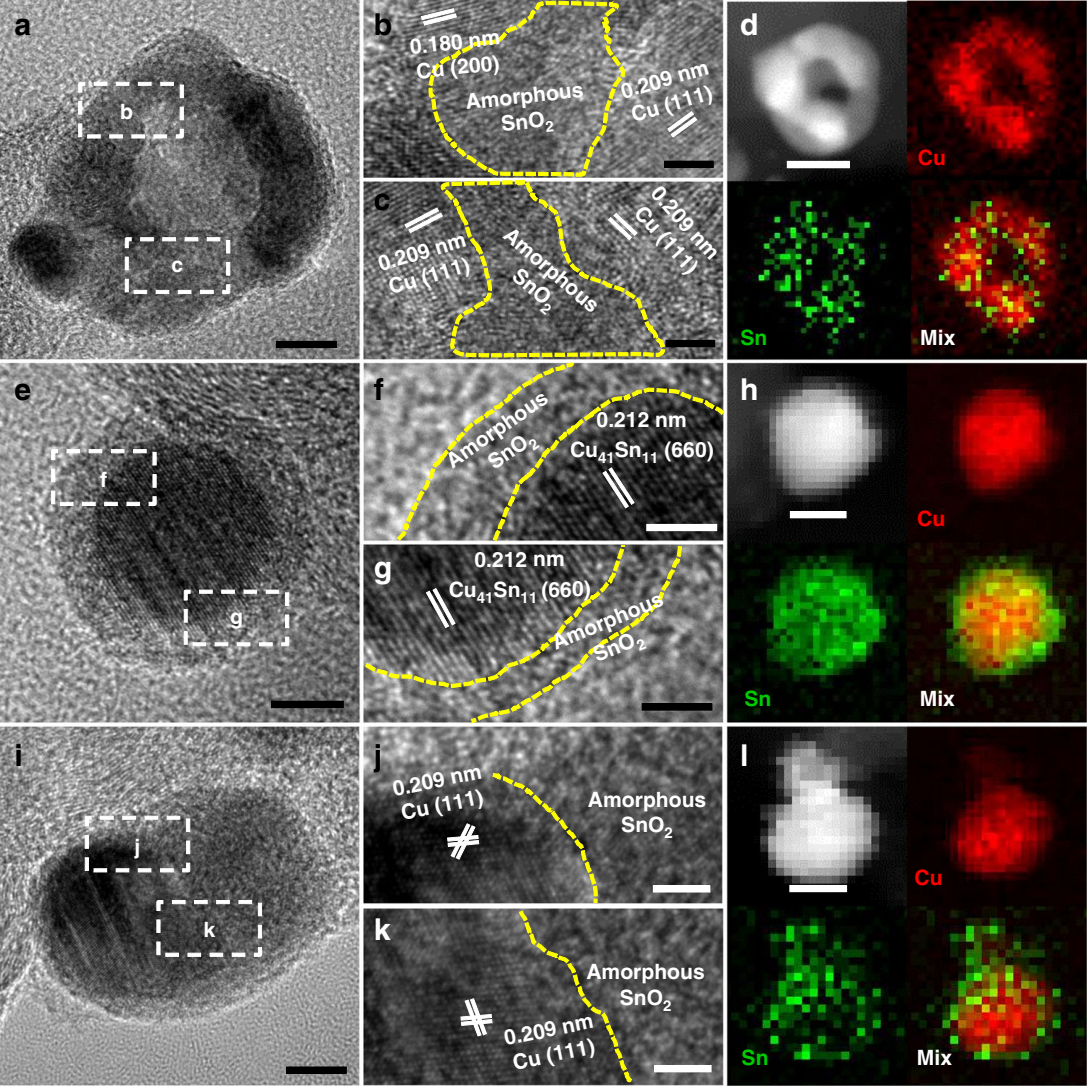
Structure and interface characterizations of CuSn NPs/C-A-ACP, CuSn NPs/C-H-ACP, and CuSn NPs/C-AH-ACP. **a**, **e**, **i** High-resolution transmission electron microscopy (HRTEM) images, **b**, **c**, **f**, **g**, **j**, **k** magnified HRTEM images, and **d**, **h**, **l** high-angle annular dark-field scanning TEM (HAADF-STEM) image and energy-dispersive X-ray spectroscopy (EDS) elemental mappings of **a**–**d** CuSn NPs/C-A-ACP, **e–h** CuSn NPs/C-H-ACP, and **i**–**l** CuSn NPs/C-AH-ACP. Scale bars, 10 nm in **d**, **h**, **l**, 5 nm in **a**, **e**, **i**, 2 nm in **b**, **c**, **f**, **g**, **j**, **k**

It is generally believed that the chemical state and structure of catalysts would gradually undergo changes during the CO_2_RR process, which can largely determine their ultimate performance. To this end, the TEM images and XPS characterizations of CuSn NPs/C-A, CuSn NPs/C-H, and CuSn NPs/C-AH after the 15 min chronoamperometry test at −0.7 V_RHE_ (denoted as CuSn NPs/C-A-ACP, CuSn NPs/C-H-ACP, and CuSn /C-AH-ACP) were carried out to investigate their evolved surface states and structures. As shown in Fig. 5a, while the CuSn NPs/C-A-ACP still maintained the hollow structure during the CO_2_RR, the lattice fringes measured in the enlarged image are 0.209 and 0.180 nm, corresponding to the Cu (111) and Cu (200) planes, respectively, being consistent with the XRD result (Supplementary Fig. 22a), suggesting that the oxidized Cu^2+^ have been reduced into metallic Cu during the CO_2_RR process (Fig. 5b, c). The chemical state changes of Cu can also be confirmed by the XPS, where the ratio of Cu^0,1+^/Cu^2+^ in the CuSn NPs/C-A-ACP increased to 2.7/1, in contrast with 0 in the CuSn NPs/C-A (Supplementary Fig. 23). As opposed to Cu, Sn was still in the form of the amorphous SnO_2_ combined with the small composition loss in the CuSn NPs/C-A-ACP (Fig. 5b, c and Supplementary Fig. 24a). The element mapping further reveals that the amorphous SnO_2_ was mixed with Cu in the CuSn NPs/C-A-ACP, which can produce numerous Cu/SnO_2_ interfaces (Fig. 5d). In contrast, the CuSn NPs/C-H-ACP maintains the Cu_41_Sn_11_@SnO_2_ core–shell structure of the initial CuSn NPs/C-H with small composition change, as confirmed by the HRTEM image, HAADF EDS elemental mappings, XRD, and EDS results (Fig. 5e–h, Supplementary Fig. 22b, and Supplementary Fig. 24b), indicating that there are hardly any Cu/SnO_2_ interfaces exposed. The structure of CuSn NPs/C-AH-ACP also undergoes a significant change, where the original hollow amorphous SnO_2_ shrinks into a solid particle combined with the Cu in the CuSn NPs/C-AH-ACP due to the element corrosion and migration during the CO_2_RR process (Fig. 5i, Supplementary Fig. 22c, and Supplementary Fig. 24c). The enlarged HRTEM and element mapping images further show that the newly evolved structure of CuSn NPs/C-AH-ACP has a few interface between Cu and SnO_2_ (Fig. 5j–l, Supplementary Fig. 25). Moreover, the surface composition of the above catalysts was also confirmed by XPS results (Supplementary Table 2). The ratios of Cu/Sn in CuSn NPs/C-H-ACP and CuSn NPs/C-AH-ACP calculated by the XPS results are lower than those by EDS results, suggesting that the Sn is mainly located on the surface in these catalysts, being consistent with the results of element mapping. Moreover, according to the XPS results the ratio of Cu/Sn in CuSn NPs/C-A-ACP is similar with the CuSn NPs/C-AH-ACP, indicating that the composition effect on the FE_CO_ improvement of CuSn NPs/C-A-ACP and CuSn NPs/C-AH-ACP is negligible. Thus, with the surface state and structure evolutions of different catalysts, the greatest difference among them is the density of the newly formed Cu/SnO_2_ interfaces, which follows the sequence: CuSn NPs/C-A-ACP > CuSn NPs/C-AH-ACP > CuSn NPs/C-H-ACP. Therefore, it is reasonable to conclude that the newly produced Cu/SnO_2_ interfaces are the active sites for improving the catalytic selectivity in those Cu–SnO_2_ bimetallic catalysts and resulting in the different selectivity for the CO_2_RR. To further prove it, the mixed Cu NPs/C and SnO_2_ NSL/C with similar Cu/Sn ratio but limited Cu/SnO_2_ interfaces were used as the catalyst for the CO_2_RR (Supplementary Fig. 26). It is shown that the FE_CO_ of mixed Cu NPs/C and SnO_2_ NSL/C at −0.7 V_RHE_ is 31.3% (Supplementary Fig. 27), which is between the FE_CO_ of CuSn NPs/C-H and of CuSn NPs/C-AH. Based on the TEM and HRTEM observation and the CO_2_RR results of various catalysts, we can conclude that the FEs_CO_ of different catalysts are systematically controlled by the Cu/SnO_2_ interfaces (Supplementary Fig. 28), confirming the importance of Cu/SnO_2_ interfaces in boosting the product selectivity of CO_2_RR.

**Fig. 6:**
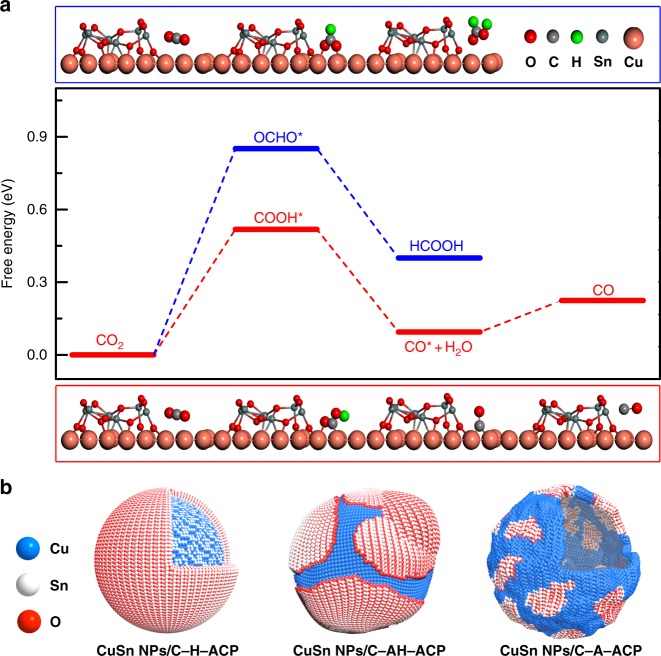
Density functional theory simulations of the CO_2_ reduction reaction on Cu/SnO_2_ interfaces. **a** Free energy profiles of two pathways for CO_2_ electroreduction on Cu/SnO_2_ interfaces. The upper and lower images are optimized geometric structures of various states (COOH*, OCHO*, and CO*) of the process on Cu/SnO_2_ interfaces, respectively. H, C, O, Cu, and Sn atoms are represented by green, gray, red, reddish brown, and dark gray spheres, respectively. **b** The models of CuSn NPs/C-A-ACP, CuSn NPs/C-H-ACP, and CuSn NPs/C-AH-ACP with different Cu/SnO_2_ interfaces

To further elucidate the Cu/SnO_2_ interfaces effect on product selectivity for Cu–SnO_2_ bimetallic catalysts, the density functional theory (DFT) calculations of Cu–SnO_2_ heterostructures were carried out. In general, the CO_2_ electroreduction usually initiates with the proton-coupled electron transfer process, then the C or O atom protonation would result in generating OCHO* or COOH* species, respectively. The adsorption free-energies of CO_2_RR intermediates and the Δ*G* of elementary steps for Cu (111) and SnO_2_ (110) surfaces were first calculated employing the computational hydrogen electrode (CHE) model^23^. As shown in Supplementary Fig. 29, the formation of OCHO* species (Δ*G* = 0.20 eV) is energetically more favorable than that of COOH* species (Δ*G* = 0.71 eV) on the Cu (111) surface. Similarly, the SnO_2_ (110) surface also favors HCOOH as the final product since the formation of OCHO* (Δ*G* = 1.48 eV) species is energetically much more favorable than that of COOH*species (Δ*G* = 2.32 eV) (Supplementary Fig. 30). Furthermore, the Cu/SnO_2_ interfacial effect on the CO_2_RR electrocatalytic performance of the Cu–SnO_2_ heterostructure were explored. The Cu/SnO_2_ interface was constructed by binding the Sn_6_O_12_ cluster onto the Cu (111) slab. Note that the surface-supported small oxide clusters are effective to describe catalytic activity of the complex^36,37^. As shown in Fig. 6a, in sharp contrast to individual Cu (111) and SnO_2_ (110) surfaces, the O atom rather than the C atom of the CO_2_ molecule prefers to be first hydrogenated at the Cu/SnO_2_ interface. Specifically, the Δ*G* for the COOH* species formation is 0.52 eV at Cu/SnO_2_ interfaces, being lower than those of 0.71 and 2.32 eV at the Cu (111) surface and SnO_2_ (110) surface, respectively. Meanwhile, the COOH* species formation is energetically more favorable than that of OCHO* species (Δ*G* = 0.85 eV) at Cu/SnO_2_ interfaces. The COOH* intermediate can be easily converted to a removed H_2_O molecule and an adsorbed CO* species with a Δ*G* of 0.43 eV. Finally, the CO* species can be desorbed easily with a ΔG of −0.13 eV to form the CO. The calculations vividly demonstrated that the construction of the Cu/SnO_2_ interface can essentially tune the catalytic products and enhance the activity of Cu–SnO_2_ bimetallic catalysts to generate CO, which are in good agreement with experimental observations. Thus owing to the more evolved Cu/SnO_2_ interfaces than that of the CuSn NPs/C-AH during the CO_2_RR process (Fig. 6b), the CuSn NPs/C-A shows a higher FE_CO_ at −0.7 V_RHE_. However, for the CuSn NPs/C-H, the exposed reactive site is only SnO_2_ (Fig. 6b), resulting in both high FE_H2_ and FE_HCOOH_ values. Although Sarfaz et al. and Li et al.^28^ have also shown high FE_CO_ at lower overpotential for CO_2_RR by the CuSn complex structures, the active site for improving the CO_2_RR performance is vague. In fact, the biggest concept difference between our work and Sarfaz’s work/Li’s work is that the Sarfaz’s work/Li’s work mainly focused on controlling the Cu/Sn composition and SnO_2_ thickness to tune the CO_2_RR, while the unexplored phase/structure effects of Cu/Sn catalysts for enhancing CO_2_RR have been decoded in our work. This is also the main reason why we can generate both CO and HCOOH with high selectivity, while only high FE_CO_ can be obtained in Sarfaz’ work/Li’s work. By further combining the experimental characterizations with the DFT calculations, we have revealed the important role of Cu/SnO_2_ interfaces on enhancing the CO_2_RR, which makes a progress to understand the phase and structure effects for improving the CO_2_RR performance.

In summary, by simple thermal annealing of CuSn core–shell NPs in controlled conditions, we have created Cu–SnO_2_ catalysts with three distinct engineered structures and phases: the CuO/hollow SnO_2_ heterostructure of CuSn NPs/C-A, the Cu_41_Sn_11_@SnO_2_ core–shell structure of CuSn NPs/C-H, and the Cu NPs/hollow SnO_2_ Janus structure of CuSn NPs/C-AH. Benefitting from the abundant Cu/SnO_2_ interfaces involved in the CuO/hollow SnO_2_ heterostructure during the electrochemical process, the CuSn NPs/C-A exhibits promising electrocatalytic performance for CO_2_RR due to the decreased Δ*G* for the formation of COOH* species at Cu/SnO_2_ interfaces. It shows the highest activity toward CO_2_RR in all the Cu–SnO_2_ heterostructures, with partial current densities of CO at −0.7 V_RHE_ over 5.9 and 5.3 times higher and of HCOOH at −1.0 V_RHE_ over 33.2 and 3.4 times higher than those of the Cu NPs/C and SnO_2_ NSL/C, respectively. Particularly, the CuSn NPs/C-A can tune the products from CO to HCOOH with an FE at approximately 70% by simply changing the electrolysis potentials. These Cu–SnO_2_ bimetallic catalysts also exhibit excellent durability after the long-term chronoamperometry test at −0.7 V_RHE_. The present study highlights a phase and structure-engineering strategy for creating high-performance electrocatalysts for the CO_2_RR and beyond.

## Methods

### Chemicals

Copper (II) acetylacetonate (Cu(acac)_2_, 97%), AA (AR), and oleylamine (CH_3_(CH_2_)_7_CH=CH(CH_2_)_7_CH_2_NH_2_, 70%) were all purchased from Sigma-Aldrich. Potassium bicarbonate (KHCO_3_, AR) was purchased from Sinopharm Chemical Reagent Co. Ltd. (Shanghai, China). Dibutyltin bis(2,4-pentanedionate) (C_18_H_32_O_4_Sn) was purchased from Strem Chemical. Nitrogen (N_2_, 99.999%) and carbon dioxide (CO_2_, 99.999%) were purchased from WuGang Gas Co. Ltd. (Shanghai, China). All the above chemicals were directly used without further purification. The water (18 MΩ*cm) used in all experiments was prepared with an ultra-pure purification system.

### Preparation of CuSn NPs

In a typical preparation of CuSn NPs, 13.3 mg Cu(acac)_2_, 6 μL C_18_H_32_O_4_Sn, 27 mg AA, and 5 mL oleylamine were mixed and ultrasonicated for approximately 0.5 h in a sealed vial (30 mL). Then the vial was transferred into an oil bath and heated at 180 °C for 3 h. The resulting colloidal products in the vial were collected by centrifugation and washed with an ethanol/cyclohexane mixture to obtain CuSn NPs.

### Preparation of CuSn NPs/C, CuSn NPs/C-A, CuSn NPs/C-H, and CuSn NPs/C-AH

In a typical preparation of CuSn NPs/C, CuSn NPs were mixed with VC-X72 carbon in 10 mL cyclohexane and sonicated for 1 h to deposit them on carbon. The products were separated by centrifugation and washed with cyclohexane/ethanol to generate CuSn NPs/C. The CuSn NPs/C was annealed in air at 250 °C for 1 h and 5% H_2_ +95% N_2_ mixed gas at 250 °C for 1 h to obtain CuSn NPs/C-A and CuSn NPs/C-H, respectively. The CuSn NPs/C-A sample was further annealed in 5% H_2_ +95% N_2_ at 250 °C for another 1 h to generate CuSn NPs/C-AH. The mass loadings in all the catalysts were fixed to be 10%+0.5%, as analyzed by inductively coupled plasma atomic emission spectroscopy (ICP-AES).

### Preparation of Cu NPs and Cu NPs/C

The preparation of Cu NPs was similar to that of CuSn NPs but without adding C_18_H_32_O_4_Sn. The resulting products were collected by centrifugation and washed with an ethanol/cyclohexane mixture. The obtained Cu NPs were then mixed with VC-X72 carbon in 10 mL cyclohexane and sonicated for 1 h to deposit them on carbon. The products were separated by centrifugation and washed with cyclohexane/ethanol to generate Cu NPs/C.

### Preparation of SnO_2_ NSL/C

In a typical preparation of SnO_2_ NSL/C, 10 mg CuSn NPs/C-A were mixed with 8 mL ethanol and 2 mL acetic acid and ultrasonicated for 1 h to remove the CuO. The products were separated by centrifugation and washed with ethanol to generate SnO_2_ NSL/C.

### Characterization

TEM and HAADF-STEM were conducted on an FEI Tecnai F20 transmission electron microscope with acceleration voltage of 200 kV. The samples were prepared by dropping samples dispersed in cyclohexane or ethanol onto carbon-coated copper TEM grids using pipettes and were dried under ambient conditions. XRD patterns were collected on the X’Pert-Pro MPD diffractometer (Netherlands PANalytical) with a Cu Kα X-ray source (*λ* = 1.540598 Å). The concentrations of catalysts were determined by ICP-AES (710-ES, Varian). XPS was performed with an SSI S-Probe XPS Spectrometer. The carbon peak at 284.6 eV was used as a reference to correct for charging effects.

### Electrochemical measurements

The electrochemical measurements of CO_2_RR was performed by a three-electrode system with a carbon paper (Toray, 1 × 1 cm^2^) as working electrode, a micro Ag/AgCl electrode (4.0 M KCl) as reference electrode, and a Pt wire as the reference electrode. In the preparation of working electrode, 8 mg catalyst was ultrasonicated with 380 µL of ethanol, 20 µL of water, and 5 µL of a 5 wt% Nafion solution for 1 has catalyst ink. Then 100 µL of the catalyst ink was dropped onto carbon paper and aired to obtain the working electrodes. The electrochemical measurements was conducted in an H-cell reactor with an anion exchange membrane (Nafion 117) on CHI660 (Chenhua, Shanghai) electrochemical workstation. Each chamber of the H-cell contained 60 mL of 0.1 M KHCO_3_ aqueous solution with an ~40 mL headspace. For the electrochemical measurements, the CO_2_ was delivered into the cathodic compartment (directly connected to the gas chromatograph (GC Agilent 7890B)) at a constant rate of 20 sccm and was allowed to purge for 30 min prior to the beginning of experiments. The gas-phase composition was analyzed by a GC equipped with a PLOT MolSieve 5A column and a Q-bond PLOT column every 15 min with different potentials applied. Liquid products were analyzed by ^1^H NMR on Agilent 600 MHz DirectDrive2 spectrometers. All potentials were given against the reversible hydrogen electrode (RHE), calculated using the Nernst equation, and the readouts were recorded with 90% Ohmic iR drop correction. The FE for the products was calculated as follows:

1 $${\mathrm{FE}} = e{\mathrm{F}} \times n/Q$$

where *e* is the number of electrons transferred for different products, *Q* is the total charge, *n* is the total amount of different product (in moles), and F is the Faraday constant.

### Computational method

DFT calculations were performed using the plane-wave technique implemented in the Vienna ab initio simulation package^38,39^. The ion–electron interaction was described using the projector-augmented plane wave approach^40^. The generalized gradient approximation expressed by the Perdew–Burke–Ernzerhof functional^41^ and a 360-eV cutoff for the plane-wave basis set were adopted in all the computations. To simulate the Cu/SnO_2_ interfaces, we placed a Sn_6_O_12_ cluster on a 5×5×3 Cu (111) surface with a 19-Å vacuum between the slabs. The Sn_6_O_12_ cluster was built based on rutile SnO_2_ bulk crystal and resembles the SnO_2_ (110) surface structure. For comparison, a 4×4×3 Cu (111) slab with a 3×3×1 *k*-point sampling was used. The SnO_2_ (110) surface was modeled by a six atomic layer, and the top three atomic layers were relaxed while the other layers were fixed at the bulk lattice position. For Cu–SnO_2_ and the Cu (111) slab, the upper most layer (including the Cu (111) slab and Sn_6_O_12_ cluster) were allowed to relax, except for those of the bottom two layers in Cu (111), which were fixed at the bulk lattice position. The Gaussian smearing method with *σ* value of 0.1 eV was used in this system. The convergence threshold was set as 10^−4^ eV in energy and 0.08 eV Å^−1^ in force. A (2 × 2 × 1) gamma centered *k*-point was chosen for the slab calculation. The solvent effect on adsorbates was simulated using the Poissson–Boltzmann implicit solvation model with a dielectric constant of 80^42^. The effects of dipole correction for adsorbates were also included.

The CHE method has been applied to determine the Gibbs free energy of the reaction species. In this method, the electrochemical potential of an electron–proton pair (H^+^ + *e*^−^) is equal to the free energy of half of hydrogen (1/2 H_2_) at standard pressure. The CO_2_RR involving a 2*e* pathway can be described by:

2 $${\mathrm{CO}}_2\left( {\mathrm{g}} \right) + \left( {{\mathrm{H}}^ + + e^ - } \right) + \ast \to {\mathrm{OCHO}} \ast$$

3 $${\mathrm{OCHO}}^\ast + \left( {{\mathrm{H}}^ + + e^ - } \right) \to \ast + {\mathrm{HCOOH}}\left( {\mathrm{l}} \right)$$

4 $${\mathrm{CO}}_2\left( {\mathrm{g}} \right) + \left( {{\mathrm{H}}^ + + e^ - } \right) + \ast \to {\mathrm{COOH}} \ast$$

5 $${\mathrm{COOH}} \ast + \left( {{\mathrm{H}}^ + + e^ - } \right) \to {\mathrm{CO}} \ast + {\mathrm{H}}_2{\mathrm{O}} \to \ast + {\mathrm{CO}}\left( {\mathrm{g}} \right)$$

where * represents the adsorption site, while OCHO*, COOH*, and CO* represent reaction intermediates, respectively. The free energy (*G*) of each intermediate estimated at *T* = 298 K was expressed as:

6 $$G = E_{{\mathrm{DFT}}} + E_{{\mathrm{ZPE}}} - TS + \smallint C_{\mathrm{p}}{\mathrm{d}}T$$

where *E*_DFT_, *E*_ZPE_, *S*, and *C*_p_ were the electronic energy, zero point energy, entropy, and heat capacity, respectively. For adsorbed intermediates, *E*_ZPE_, *S*, and *C*_p_ were determined by vibration frequency calculations via standard methods, where all 3*N* degrees of freedom were treated as harmonic vibration motions with neglecting contributions from the slab; for molecules, however, those values were taken from the NIST database^43^. The contribution of zero point energy, entropy corrections, and heat capacity to *G* is provided in Supplementary Table 3.

## Electronic supplementary material

Supplementary Information

## Data Availability

All relevant data are available from the authors on request.
