# Supplementary material for: Phase and structure engineering of copper tin heterostructures for efficient electrochemical carbon dioxide reduction
Source: Nat Commun. 2018 Nov 22;9:4933. doi: 10.1038/s41467-018-07419-z (PMC6250663; doi:10.1038/s41467-018-07419-z)
Supplement: Supplementary file 1 — Supplementary Information [file 41467_2018_7419_MOESM1_ESM.pdf]

Supporting information

Phase and Structure Engineering of Copper Tin Heterostructures for Efficient Electrochemical Carbon Dioxide Reduction

Wang *et al.*

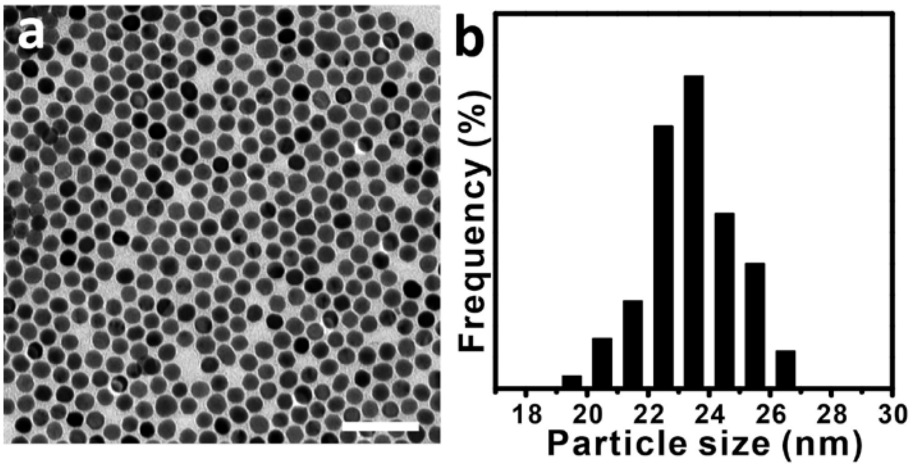

Supplementary Figure 1. Particle size distribution of CuSn NPs. **a** Transmission electron microscopy (TEM) image and **b** particle size histogram of CuSn NPs. Scale bars, 100 nm in **a**.

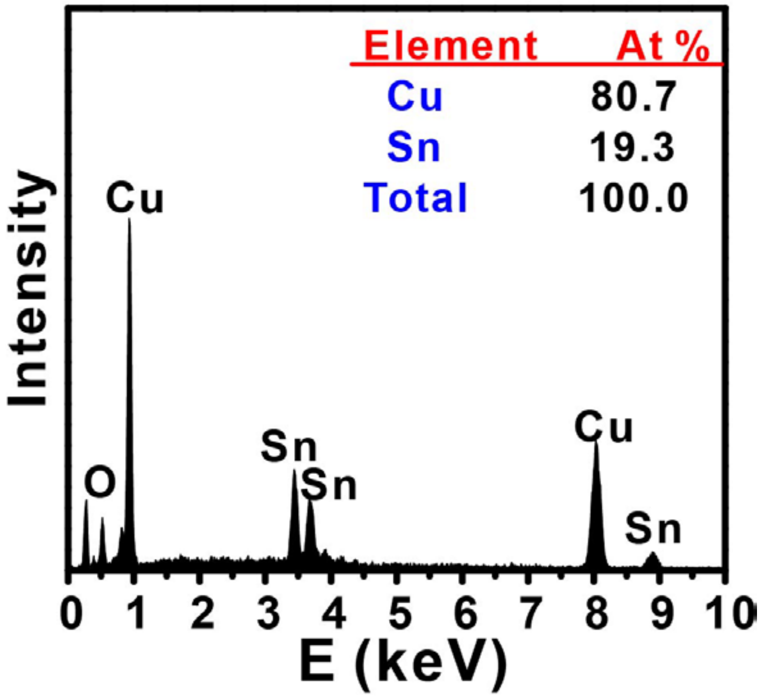

Supplementary Figure 2. Scanning electron microscopy energy-dispersive X-ray spectroscopy (SEM-EDS) of CuSn NPs.

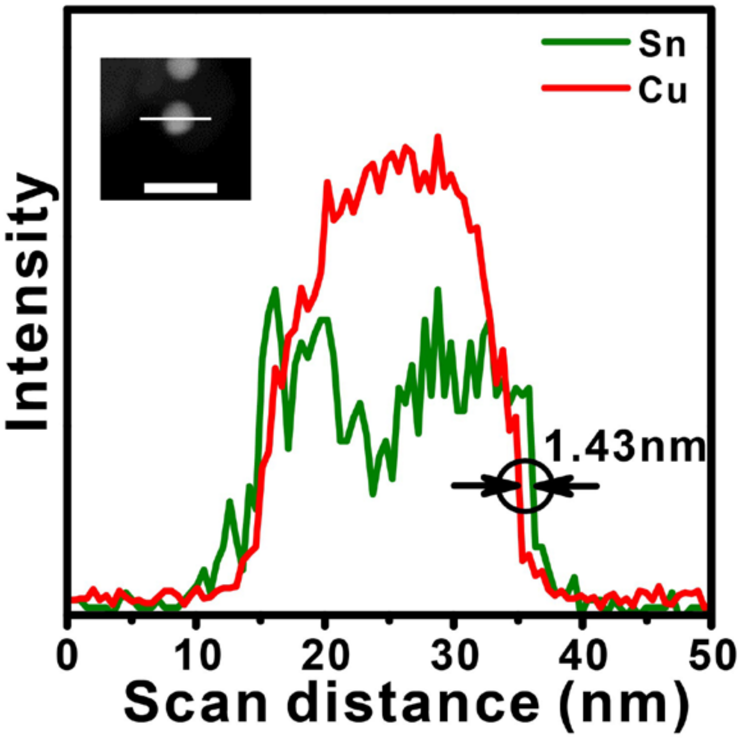

Supplementary Figure 3. High-angle annular dark-field scanning transmission electron microscopy (HAADF-STEM) line scan of CuSn NPs. Scale bars, 50 nm.

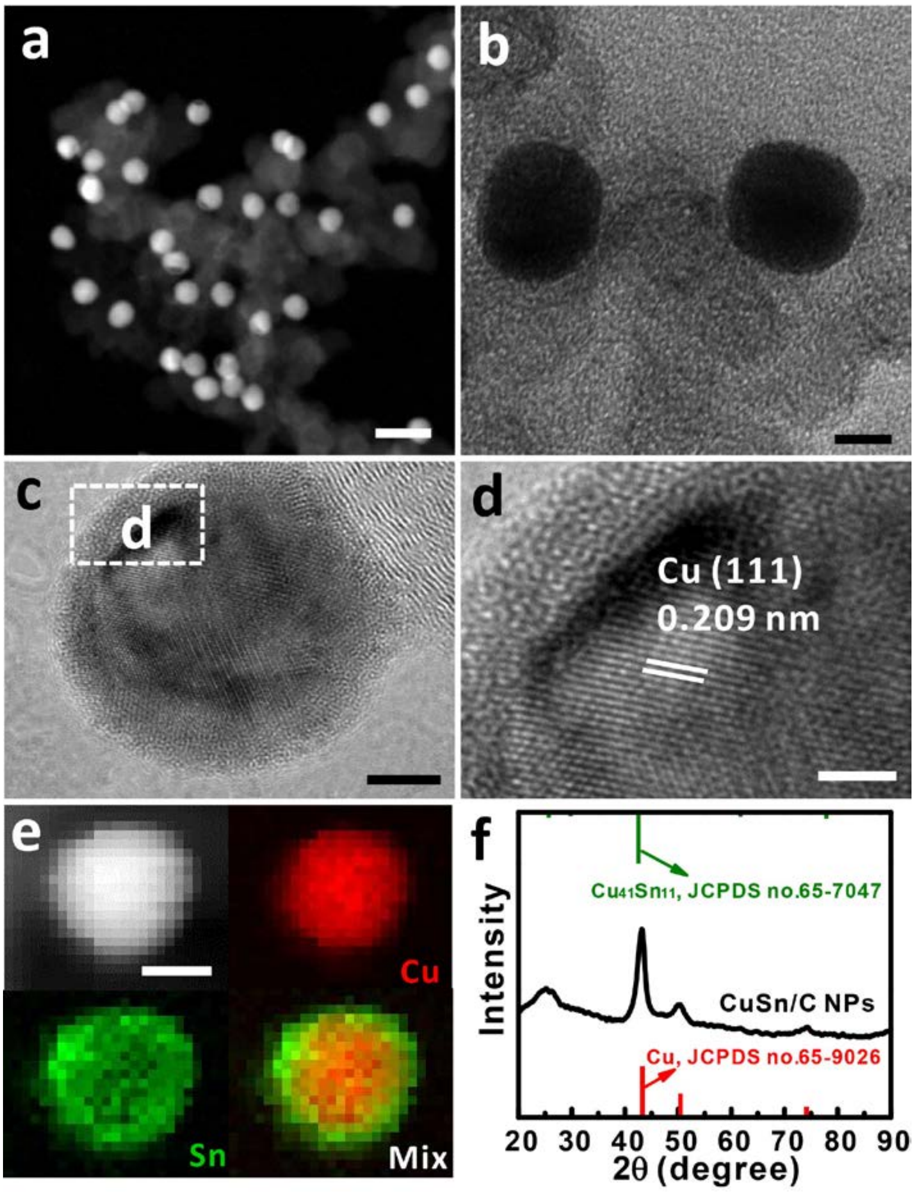

Supplementary Figure 4. Structure and phase characterization of CuSn NPs/C. **a** High-angle annular dark-field scanning TEM (HAADF-STEM) and **b** transmission electron microscopy (TEM) images of CuSn NPs/C. **c** High-resolution TEM (HRTEM) and **d** magnified HRTEM images of CuSn NPs/C. **e** HAADF-STEM image and corresponding energy-dispersive X-ray spectroscopy (EDS) elemental mappings of CuSn NPs/C. **f** X-ray diffraction (XRD) pattern of CuSn NPs/C. Scale bars, 50 nm in **a**, 10 nm in **b** and **e**, 5 nm in **c**, 2 nm in **d**.

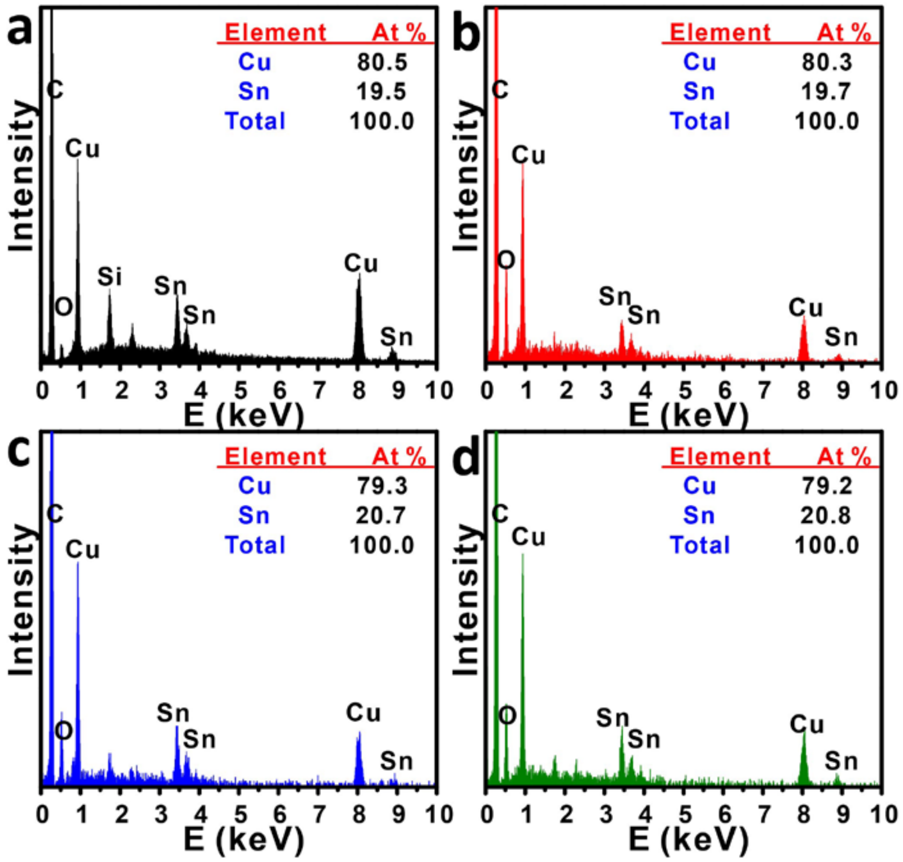

Supplementary Figure 5. Composition analysis of Cu-SnO_2 bimetallic catalysts. Scanning electron microscopy energy-dispersive X-ray spectroscopy (SEM-EDS) of **a** CuSn NPs/C, **b** CuSn NPs/C-A, **c** CuSn NPs/C-H and **d** CuSn NPs/C-AH.

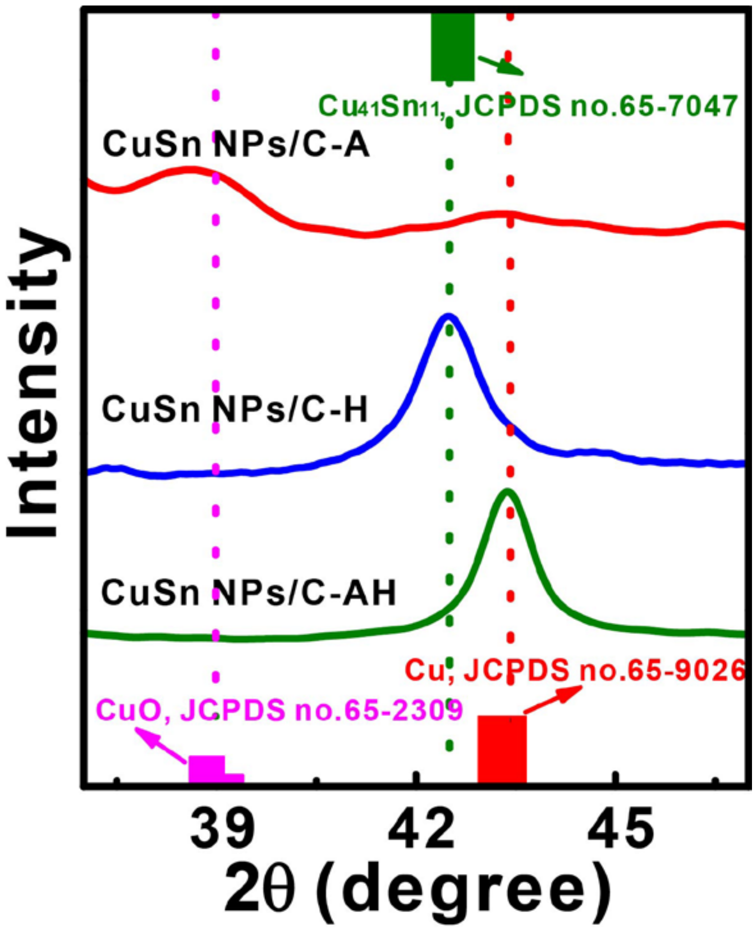

Supplementary Figure 6. The magnified X-ray diffraction (XRD) curves from the dashed region marked in **Fig. 2a**.

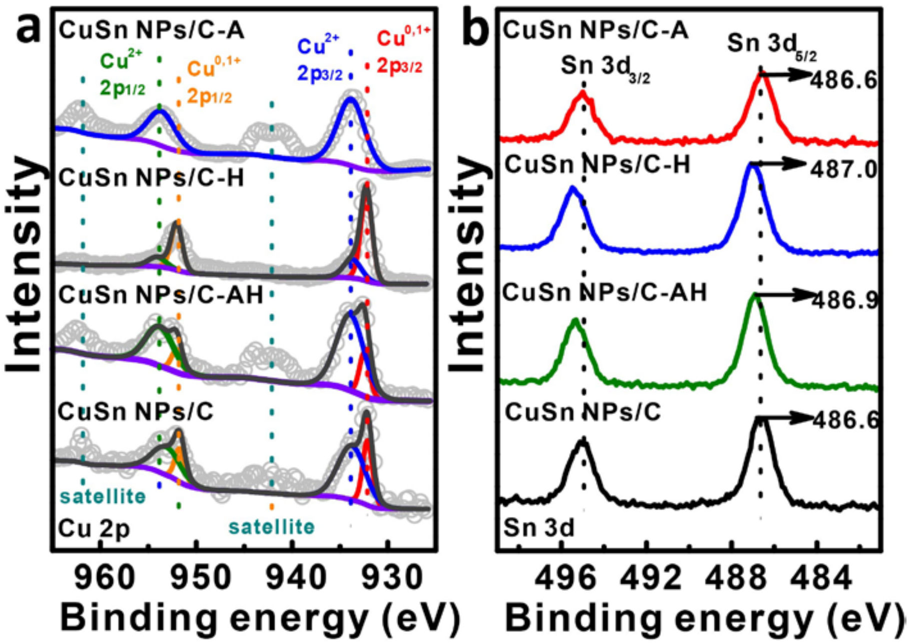

Supplementary Figure 7. Surface valence analysis of Cu-SnO_2 bimetallic catalysts. **a** Cu 2p and **b** Sn 3d X-ray photoelectron spectroscopy (XPS) curves of CuSn NPs/C, CuSn NPs/C-A, CuSn NPs/C-H and CuSn NPs/C-AH.

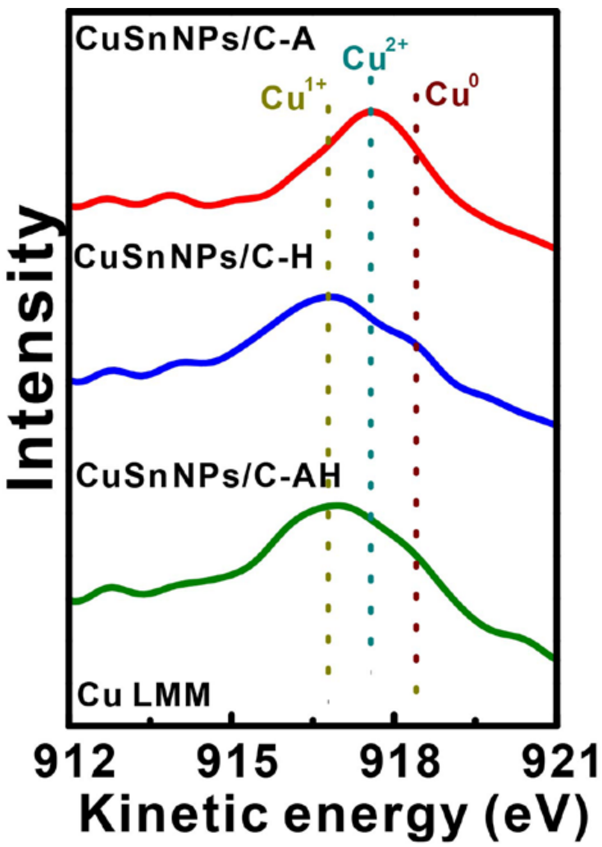

Supplementary Figure 8. Cu LMM auger spectra of CuSn NPs/C-A, CuSn NPs/C-H and CuSn NPs/C-AH.

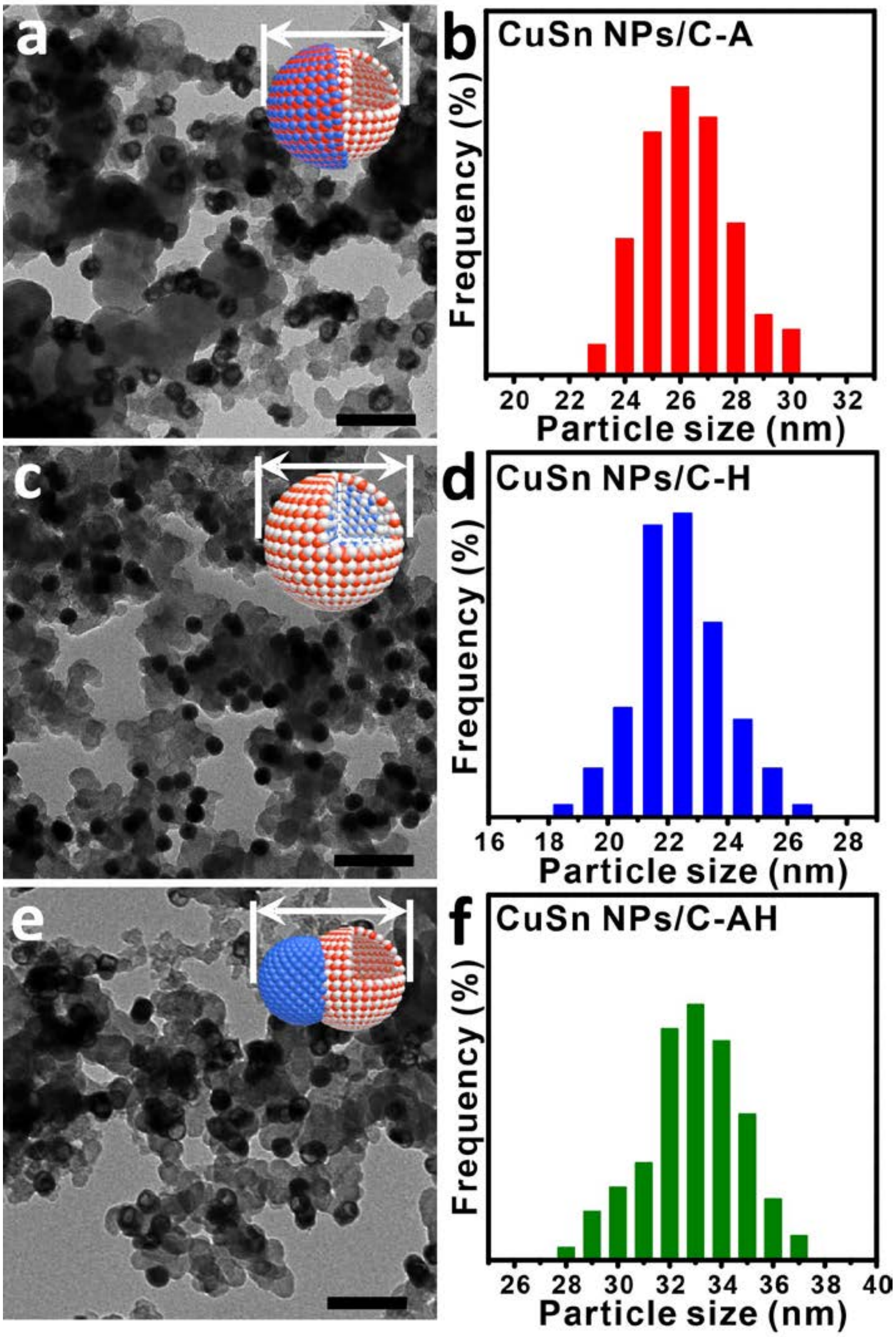

Supplementary Figure 9. Particle size distribution of Cu-SnO_2 bimetallic catalysts. **a**, **c**, **e** Transmission electron microscopy (TEM) images and **b**, **d**, **f** particle size histograms of **a**, **b** CuSn NPs/C-A, **c**, **d** CuSn NPs/C-H and **e**, **f** CuSn NPs/C-AH. Scale bars, 100 nm in **a**, **c** and **e**.

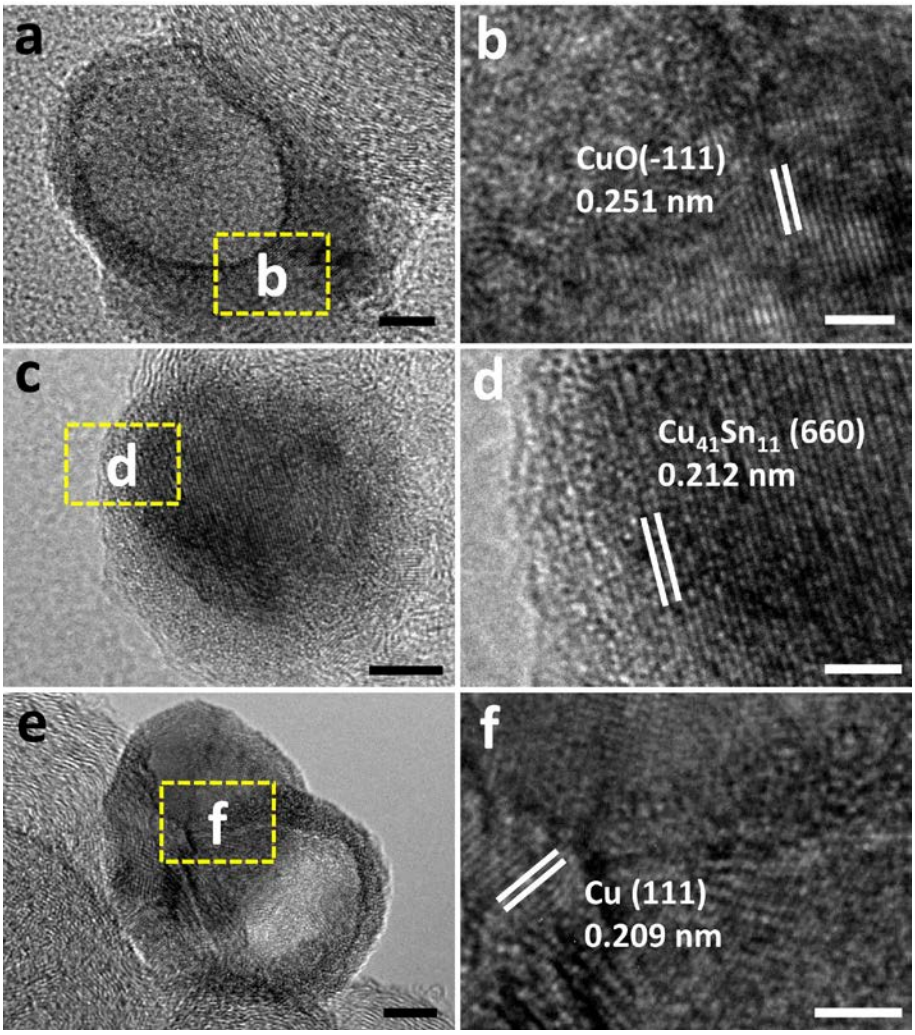

Supplementary Figure 10. **a**, **c**, **e** High-resolution transmission electron microscopy (HRTEM) and **b**, **d**, **f** magnified HRTEM images of **a**, **b** CuSn NPs/C-A, **c**, **d** CuSn NPs/C-H and **e**, **f** CuSn NPs/C-AH. Scale bars, 5 nm in **a**, **c** and **e**, 2 nm in **b**, **d** and **f**.

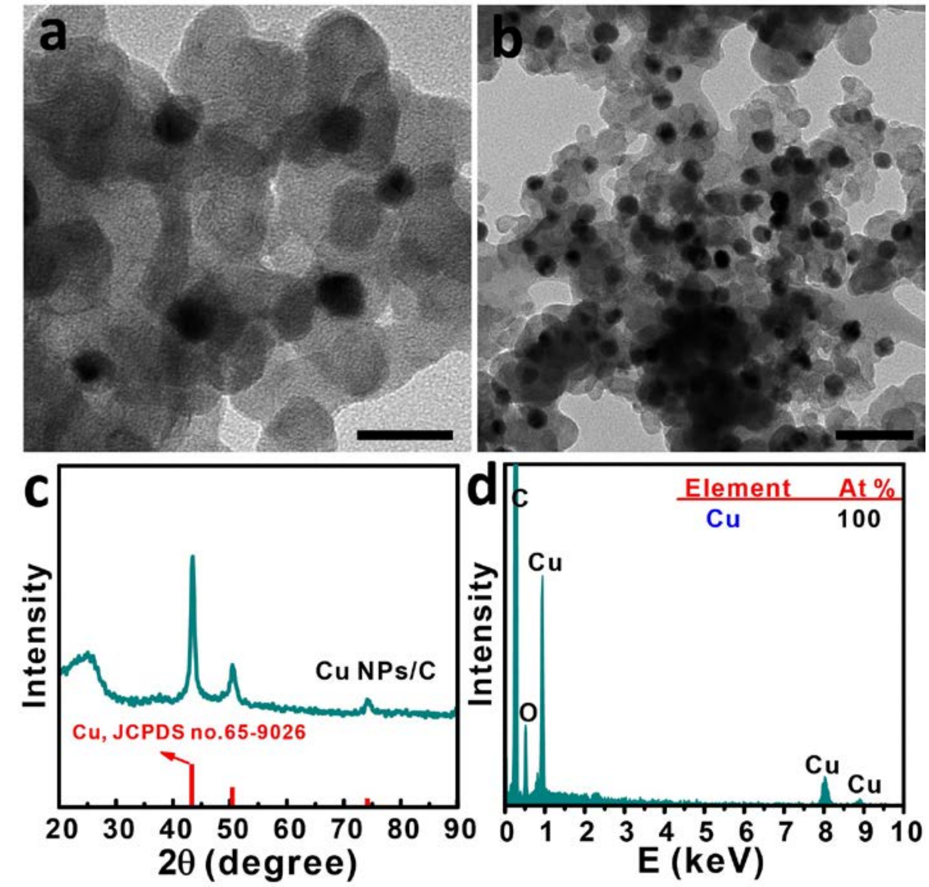

Supplementary Figure 11. Structure and phase characterization of Cu NPs/C. **a**, **b** Transmission electron microscopy (TEM) images, **c** X-ray diffraction (XRD) pattern and **d** energy-dispersive X-ray spectroscopy (EDS) pattern of Cu NPs/C. Scale bars, 100 nm in **a**, 50 nm in **b**.

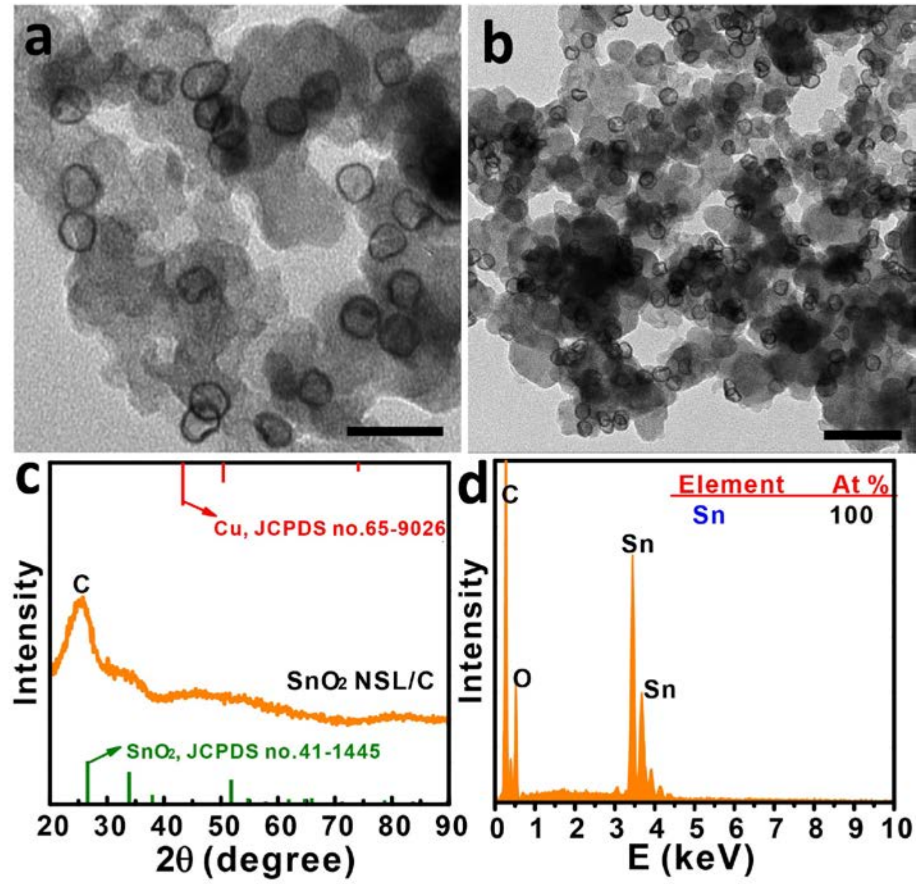

Supplementary Figure 12. Structure and phase characterization of SnO_2 NSL/C. **a**, **b** Transmission electron microscopy (TEM) images, **c** X-ray diffraction (XRD) pattern and **d** energy-dispersive X-ray spectroscopy (EDS) pattern of SnO_2 NSL/C. The peak at 25.5° in **c** belongs to the C support. Scale bars, 100 nm in **a**, 50 nm in **b**.

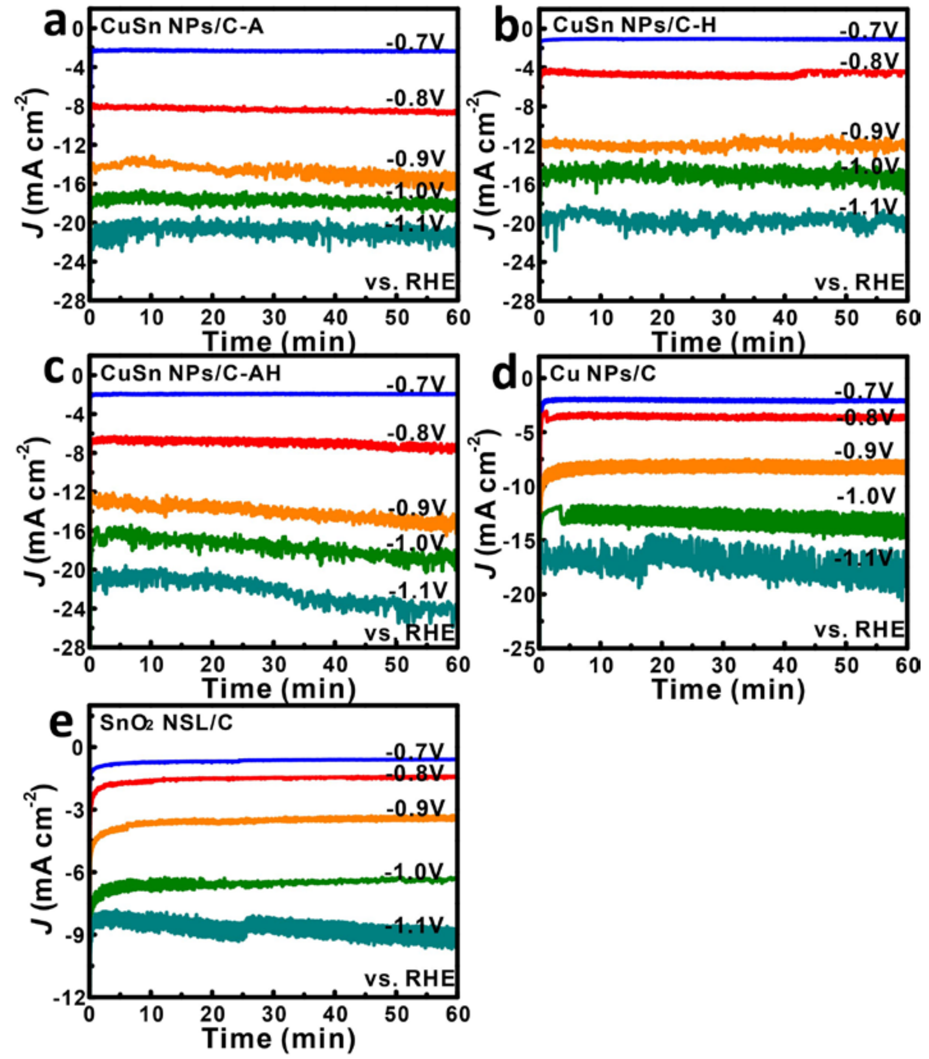

Supplementary Figure 13. Chronoamperometry results of **a** CuSn NPs/C-A, **b** CuSn NPs/C-H, **c** CuSn NPs/C-AH, **d** Cu NPs/C and **e** SnO_2 NSL/C for the electrochemical CO_2 reduction reaction.

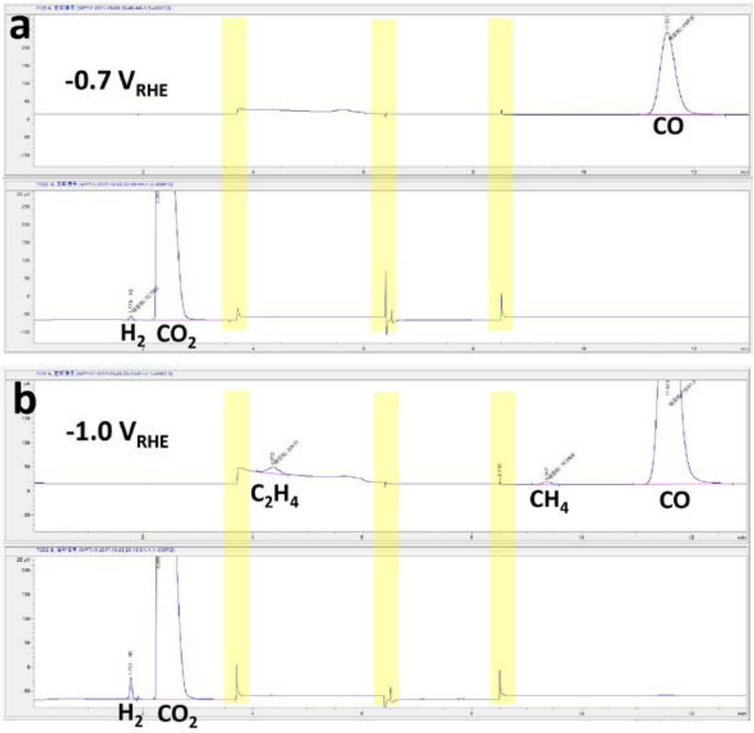

Supplementary Figure 14. Gas chromatography (GC) traces of gaseous products during the electrochemical CO_2 reduction process at **a** -0.7 V_{RHE} and **b** -1.0 V_{RHE} for the CuSn NPs/C-A. The peaks in the yellow regions are attributed to the valves switch of GC.

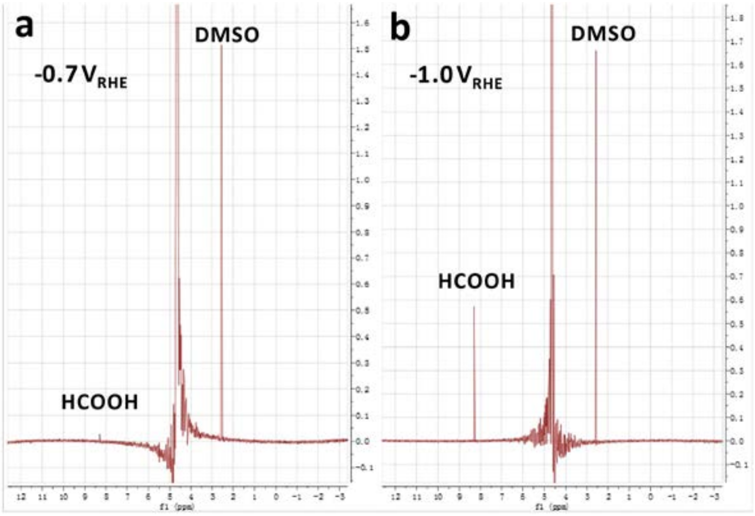

Supplementary Figure 15. ^{1}H nuclear magnetic resonance (^{1}H-NMR) spectrum of the electrolyte after electrochemical CO_2 reduction process at **a** -0.7 V_{RHE} and **b** -1.0 V_{RHE} for the CuSn NPs/C-A.

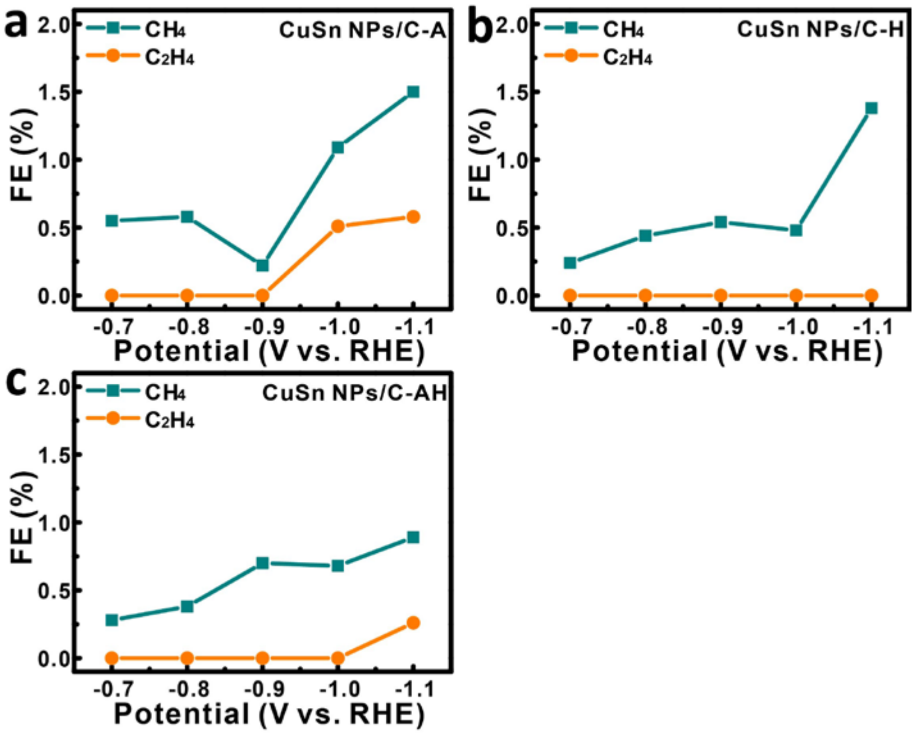

Supplementary Figure 16. Reduction potential dependent faradaic efficiencies (FEs) of CH_4 and C_2H_4 for **a** CuSn NPs/C-A, **b** CuSn NPs/C-H and **c** CuSn NPs/C-AH.

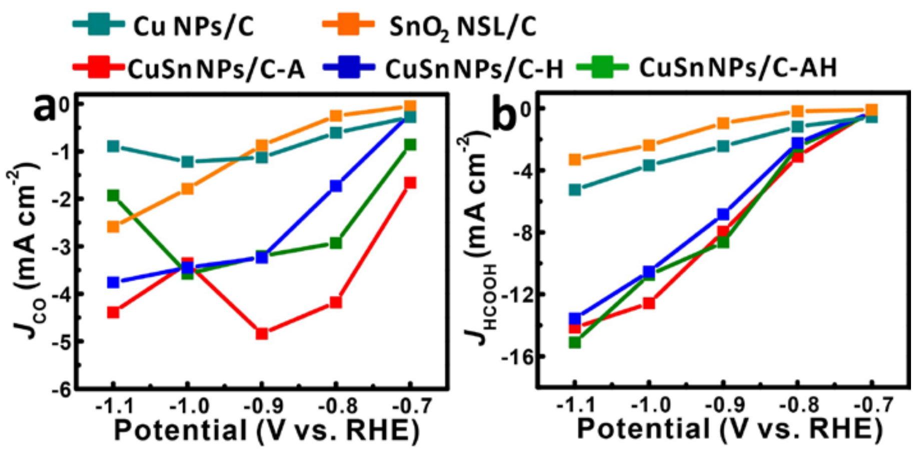

Supplementary Figure 17. Performance comparison of different catalysts for CO_2 electroreduction. **a** CO and **b** H_2 partial current densities for electrochemical CO_2 reduction reaction of CuSn NPs/C-A, CuSn NPs/C-H, CuSn NPs/C-AH, Cu NPs/C and SnO_2 NSL/C.

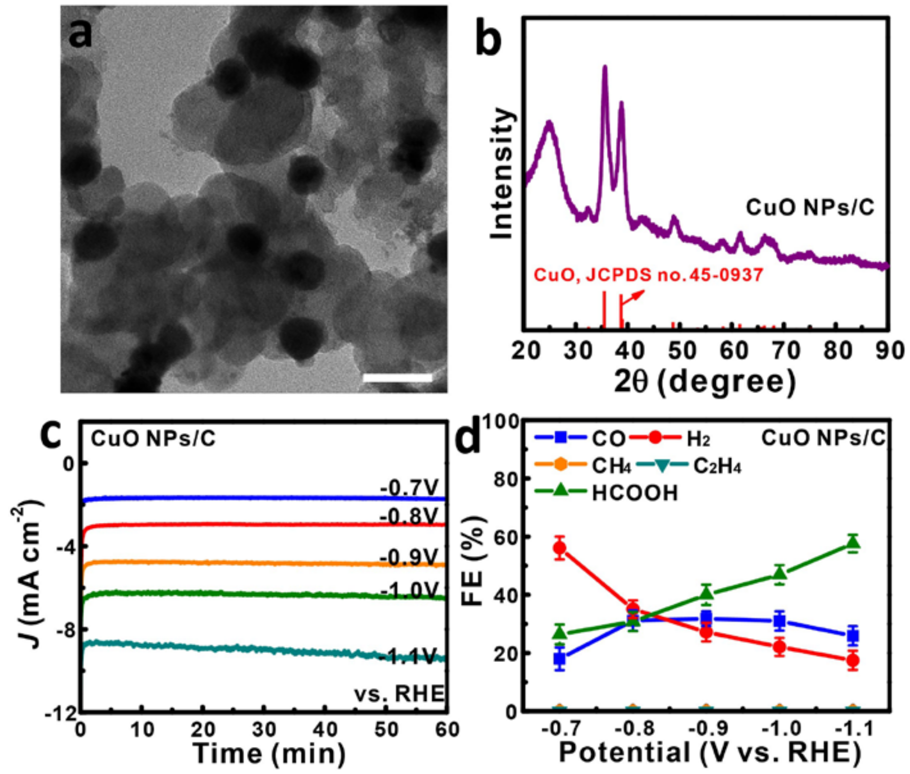

Supplementary Figure 18. Electrochemical CO_2 reduction reaction performance of CuO NPs/C. **a** Transmission electron microscopy (TEM) images, **b** X-ray diffraction (XRD) pattern, **c** chronoamperometry results and **d** reduction potential dependent products' faradaic efficiencies (FEs) of CuO NPs/C. Scale bars, 50 nm in **a**.

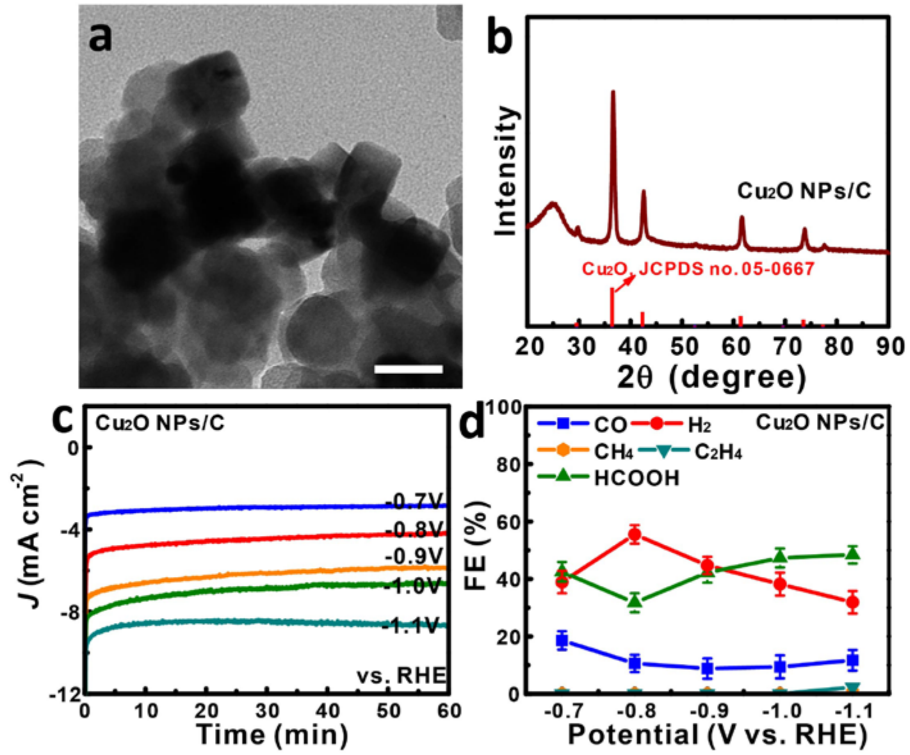

Supplementary Figure 19. Electrochemical CO_2 reduction reaction performance of Cu_2O NPs/C. **a** Transmission electron microscopy (TEM) image, **b** X-ray diffraction (XRD) pattern, **c** chronoamperometry results and **d** reduction potential dependent products' faradaic efficiencies (FEs) of Cu_2O NPs/C. Scale bars, 50 nm in **a**.

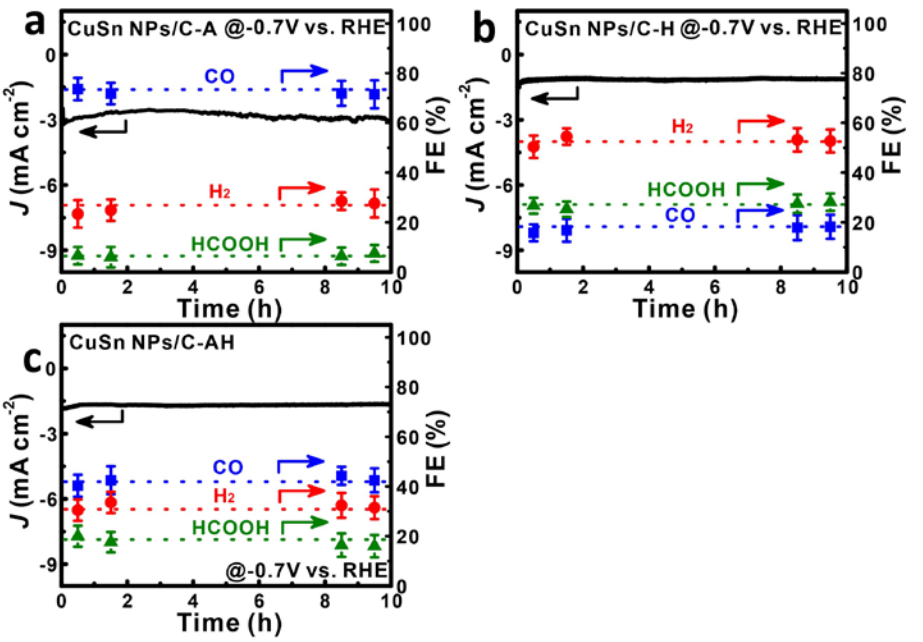

Supplementary Figure 20. Long-term stability of **a** CuSn NPs/C-A, **b** CuSn NPs/C-H and **c** CuSn NPs/C-AH for electrochemical CO_2 reduction reaction at -0.7 V_{RHE}.

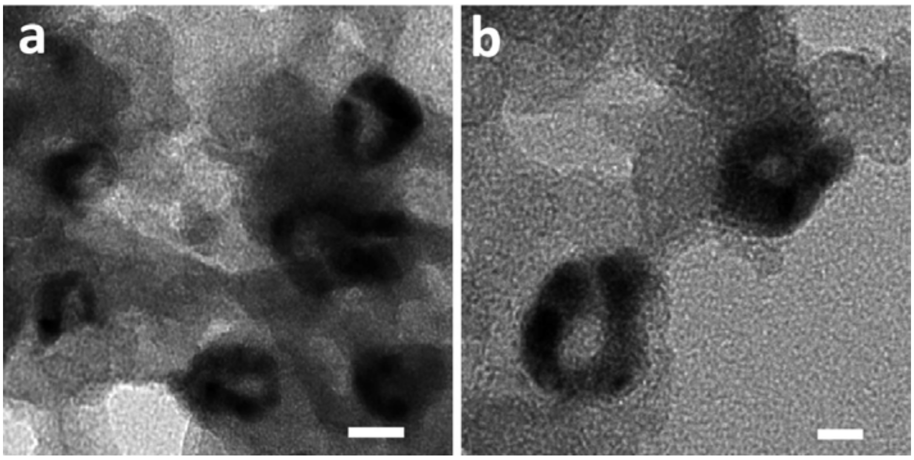

Supplementary Figure 21. Transmission electron microscopy (TEM) images of CuSn NPs/C-A after long-term stability test of electrochemical CO_2 reduction reaction at -0.7 V_{RHE}. Scale bars, 20 nm in **a**, 10 nm in **b**.

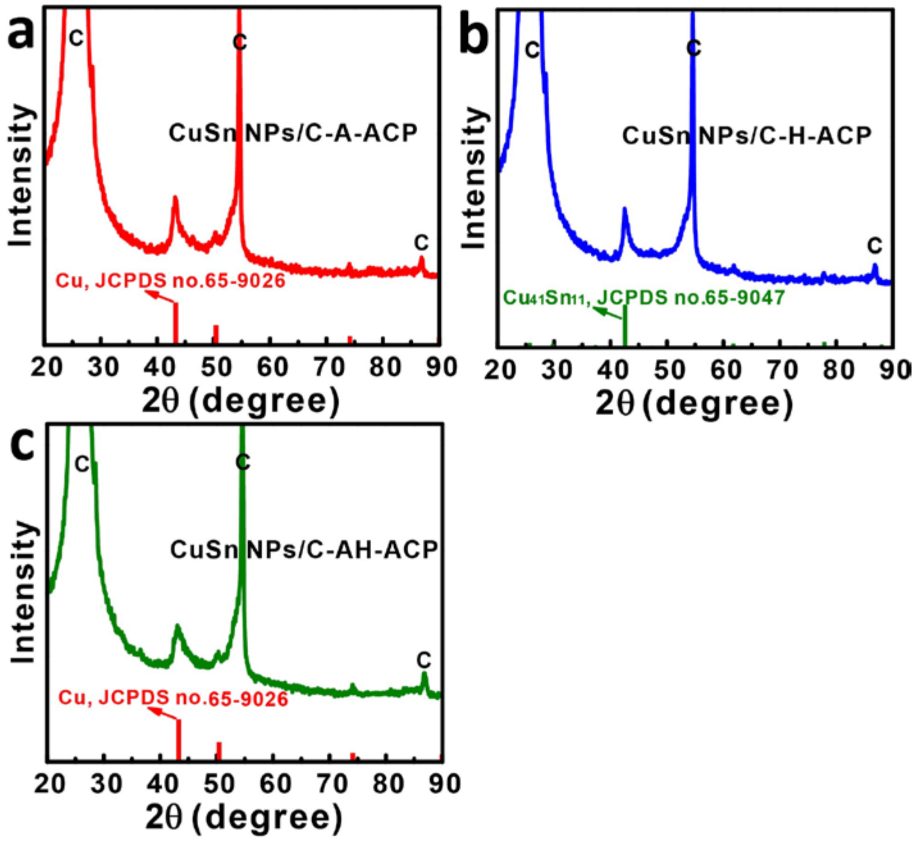

Supplementary Figure 22. Phase characterization of Cu-SnO_2 bimetallic catalysts during the CO_2 electroreduction process. X-ray diffraction (XRD) patterns of **a** CuSn NPs/C-A-ACP, **b** CuSn NPs/C-H-ACP and **c** CuSn NPs/C-AH-ACP.

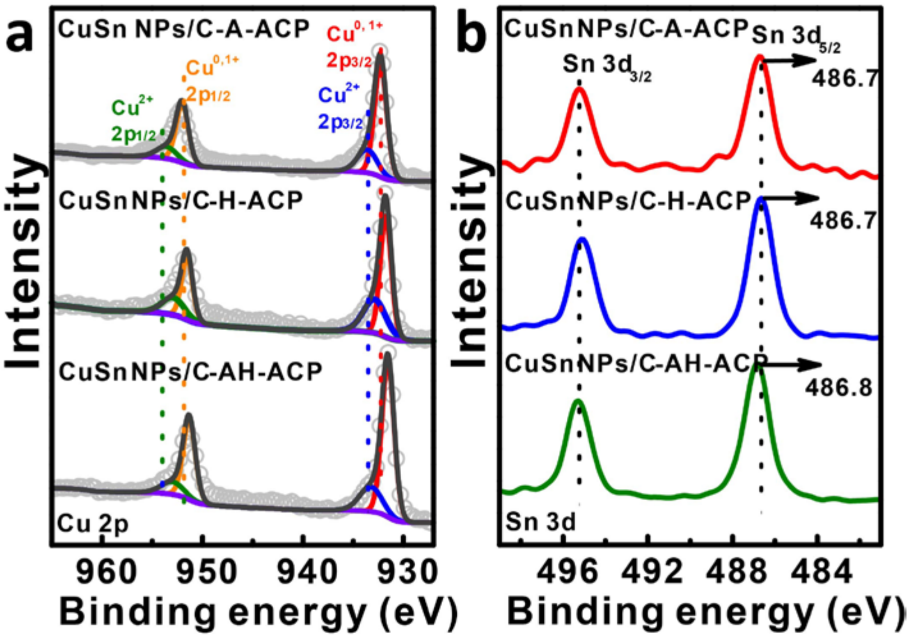

Supplementary Figure 23. Surface valence analysis of Cu-SnO_2 bimetallic catalysts during the CO_2 electroreduction process. X-ray photoelectron spectroscopy (XPS) patterns of **a** Cu 2p and **b** Sn 3d of CuSn NPs/C-A-ACP, CuSn NPs/C-H-ACP and CuSn NPs/C-AH-ACP.

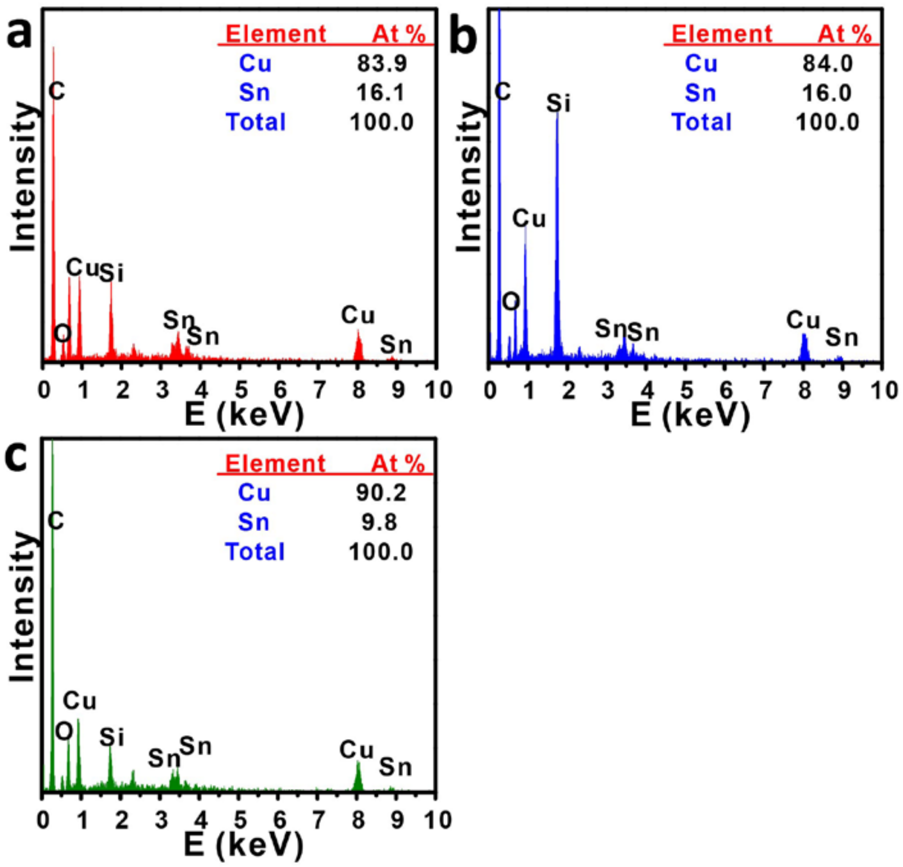

Supplementary Figure 24. Composition analysis of Cu-SnO_2 bimetallic catalysts during the CO_2 electroreduction process. Scanning electron microscopy energy-dispersive X-ray spectroscopy (SEM-EDS) of **a** CuSn NPs/C-A-ACP, **b** CuSn NPs/C-H-ACP and **c** CuSn NPs/C-AH-ACP.

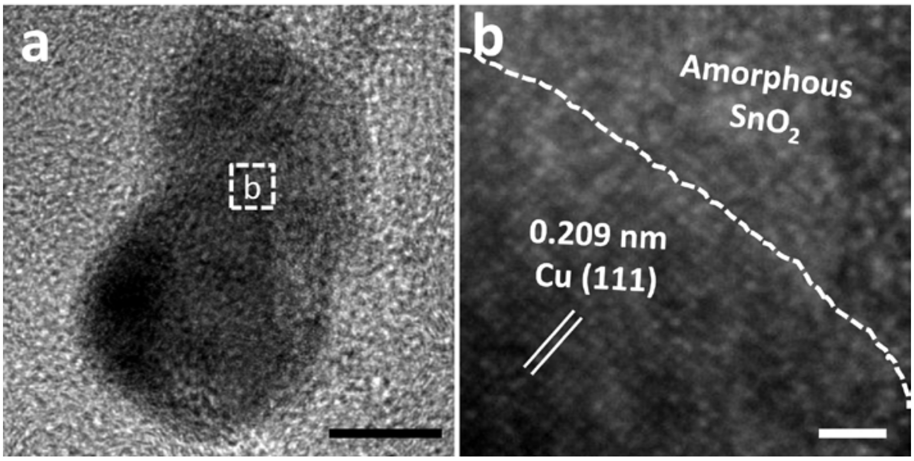

Supplementary Figure 25. a High-resolution transmission electron microscopy (HRTEM) image and **b** magnified HRTEM image recorded from regions b marked in **a** of CuSn NPs/C-AH-ACP. Scale bars, 10 nm in **a**, 1 nm in **b**.

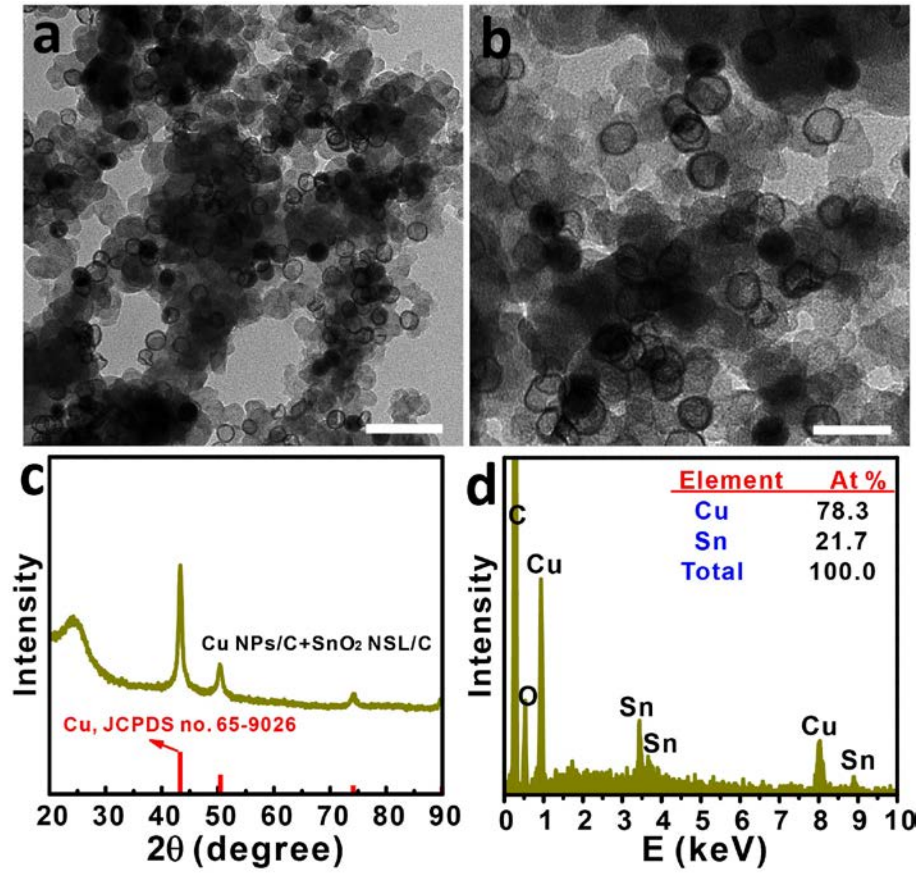

Supplementary Figure 26. Structure and phase characterization of Cu NPs/C mixed with SnO_2 NSL/C. **a**, **b** Transmission electron microscopy (TEM) images, **c** X-ray diffraction (XRD) pattern and **d** energy-dispersive X-ray spectroscopy (EDS) pattern of Cu NPs/C mixed with SnO_2 NSL/C. Scale bars, 100 nm in **a**, 50 nm in **b**.

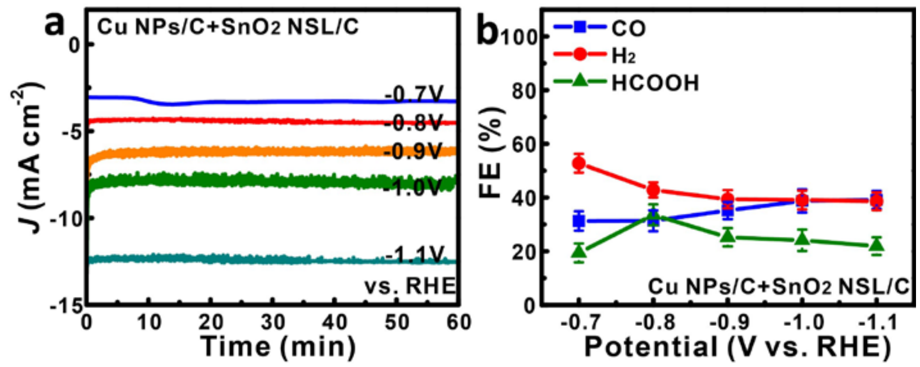

Supplementary Figure 27. Electrochemical CO_2 reduction reaction performance of Cu NPs/C mixed with SnO_2 NSL/C. **a** Chronoamperometry curves and **b** reduction potential dependent products' faradaic efficiencies (FEs) of CuSn NPs/C mixed with SnO_2 NSL/C.

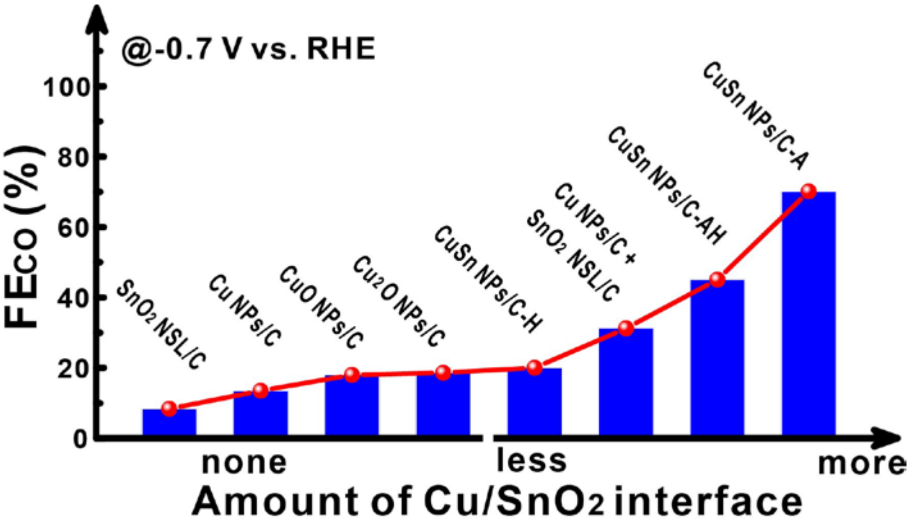

Supplementary Figure 28. The CO faradaic efficiency as function of the amount of Cu/SnO_2 interface of different catalysts at -0.7 V_{RHE} for CO_2 electroreduction.

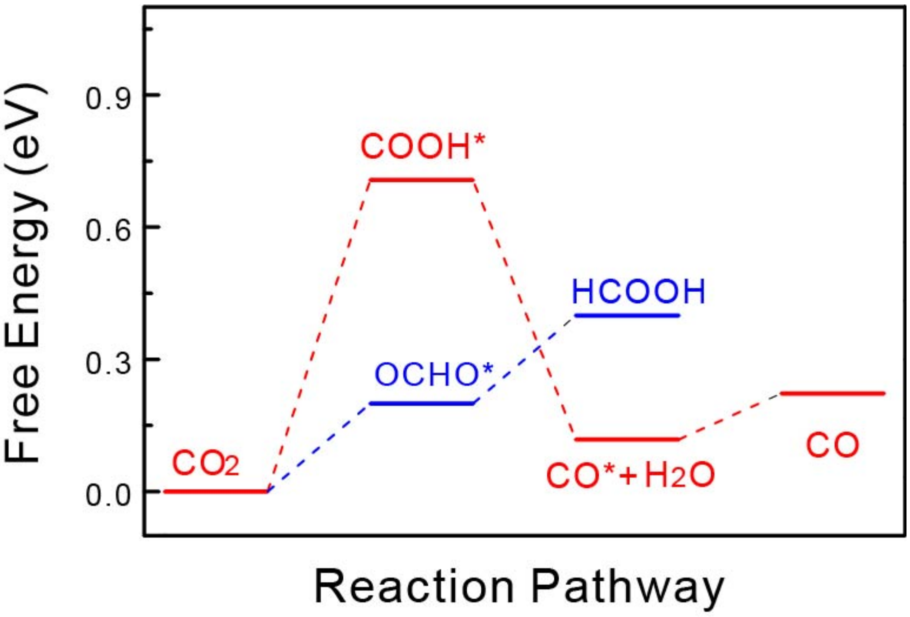

Supplementary Figure 29. Free energy profiles of two CO_2 electroreduction pathways for CO_2 electroreduction on Cu (111) surface.

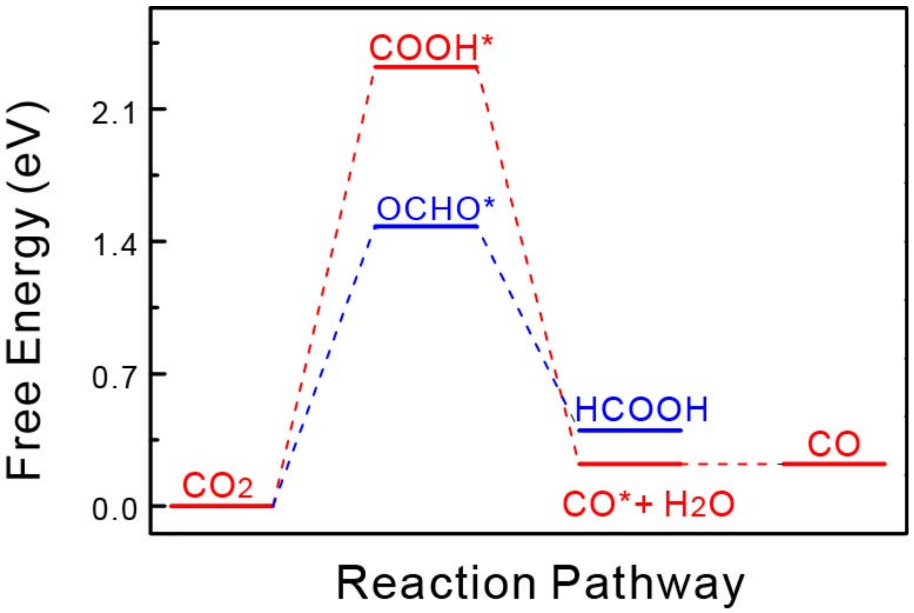

Supplementary Figure 30. Free energy profiles of two pathways for CO_2 electroreduction on SnO_2 (110) surface.

Supplementary Table 1. Summary of faradaic efficiency (%) of CO and HCOOH for Cu-based catalysts. approach to -0.7 V_{RHE} and -1.0 V_{RHE}.

Catalysts	Electrolyte	FE of products approach to -0.7 V_{RHE} (%)		FE of products approach to -1.0 V_{RHE} (%)		Reference
		CO	HCOOH	CO	HCOOH	
$AuCu_3$ NPs	0.1 M $KHCO_3$	~15	~4.5	~31	~4.2	*Nat. Commun.* **5**, 4948 (2014)
AuCu NPs		~39	~2.5	~49	~2	
Au_3Cu NPs		~65	~3	~53	~2.5	
Ordered CuPd	1 M KOH	~73	--	~57	--	*J. Am. Chem. Soc.* **139**, 47-50 (2017)
Phase-separated CuPd		~49	--	~38	--	
Disordered CuPd		~19	--	--	--	
$Cu_{90.5}Ni_{9.5}$	0.05 M $KHCO_3$	0	~8	0	~16	*J. Electrochem. Soc.* **138**, 3382-3398 (1991)
$Cu_{46.1}Pb_{53.9}$		~4	~47	~3.5	~42	
$Cu_{38}Cd_{62}$		~6	~2	~40	~7	
$Cu_{18.3}Ag_{81.7}$		~20	~13	~20	~15	
$Cu_{57}Sn_{43}$		~17	~50	~16	~57	

Cu-SnO_2 core-shell (0.8 nm thick)	0.5 M $KHCO_3$	93	~2	80	~3	*J. Am. Chem. Soc.* **139**, 4290-4293 (2017)
$Cu_{10}Zn$	0.1 M $KHCO_3$	~8	0	~1	~2	*ACS Catal.* **6**, 8239-8247 (2016)
Cu_4Zn		~8	0	~35	~1	
Cu_2Zn		~8	0	~32	0	
Ag-Cu_2O_{PB}	0.1 M $KHCO_3$	--	--	~15	~24	*ACS Catal.* **7**, 8594-8604 (2017)
Ag-Cu_2O_{PS}		--	--	~7.5	~32	
**CuSn NPs/C-A**	**0.1 M $KHCO_3$**	**70.1**	**7**	**18.7**	**71.5**	**This work**

Supplementary Table 2. The ratios of Cu/Sn in different CuSn NPs/C-ACP catalysts achieved from the energy-dispersive X-ray spectroscopy (EDS) and X-ray photoelectron spectroscopy (XPS) results.

Sample	Ratio of Cu/Sn	
	EDS	XPS
CuSn NPs/C-A-ACP	83.9/16.1	81.2/18.9
CuSn NPs/C-H-ACP	84.0/16.0	65.3/34.7
CuSn NPs/C-AH-ACP	90.2/9.8	79.5/20.5

Supplementary Table 3. Zero-pint energy correction (E_{ZPE}), entropy contribution (TS), heat capacity, and the total free energy correction ($G - E_{elec}$) in this study.

Species	E_{ZPE} (eV)	∫*CpdT (eV)*	$-TS$ (eV)	$G - E_{elec}$ (eV)
H_2	0.27	0.09	–0.42	–0.06
H_2O	0.57	0.10	–0.69	–0.02
CO	0.13	0.09	–0.61	–0.39
CO_2	0.31	0.12	–0.68	–0.25
HCOOH	0.89	0.09	–0.99	–0.01
COOH* on Cu-SnO_2	0.60	0.11	–0.25	0.46
OCHO* on Cu-SnO_2	0.61	0.11	–0.25	0.47
CO* on Cu-SnO_2	0.18	0.08	–0.13	0.13
COOH* on Cu (111)	0.58	0.12	–0.30	0.40
OCHO* on Cu (111)	0.61	0.11	-0.23	0.49
CO* on Cu (111)	0.18	0.07	–0.13	0.12
COOH* on SnO_2 (110)	0.60	0.11	–0.26	0.45
OCHO* on SnO_2 (110)	0.63	0.09	–0.16	0.56
CO* on SnO_2 (110)	0.18	0.09	–0.23	0.04
